# Gestational Diabetes Is Uniquely Associated With Altered Early Seeding of the Infant Gut Microbiota

**DOI:** 10.3389/fendo.2020.603021

**Published:** 2020-11-27

**Authors:** Taylor K. Soderborg, Charles M. Carpenter, Rachel C. Janssen, Tiffany L. Weir, Charles E. Robertson, Diana Ir, Bridget E. Young, Nancy F. Krebs, Teri L. Hernandez, Linda A. Barbour, Daniel N. Frank, Miranda Kroehl, Jacob E. Friedman

**Affiliations:** ^1^ Department of Pediatrics, Section of Neonatology, University of Colorado Anschutz Medical Campus, Aurora, CO, United States; ^2^ Division of Biostatistics and Epidemiology, University of Colorado School of Public Health, University of Colorado Anschutz Medical Campus, Aurora, CO, United States; ^3^ Department of Food Science and Human Nutrition, Colorado State University, Fort Collins, CO, United States; ^4^ Department of Medicine, Division of Infectious Disease, University of Colorado Anschutz Medical Campus, Aurora, CO, United States; ^5^ Department of Pediatrics, Section of Nutrition, University of Colorado Anschutz Medical Campus, Aurora, CO, United States; ^6^ Department of Medicine, Division of Endocrinology, Metabolism & Diabetes, University of Colorado Anschutz Medical Campus, Aurora, CO, United States; ^7^ College of Nursing, University of Colorado Anschutz Medical Campus, Aurora, CO, United States; ^8^ Department of Obstetrics and Gynecology, Division of Maternal Fetal Medicine, University of Colorado Anschutz Medical Campus, Aurora, CO, United States

**Keywords:** gestational diabetes, maternal obesity, microbiota, excess gestational weight gain, infant, short-chain fatty acids

## Abstract

Gestational diabetes mellitus (GDM) is a worldwide public health problem affecting up to 27% of pregnancies with high predictive values for childhood obesity and inflammatory diseases. Compromised seeding of the infant gut microbiota is a risk factor for immunologic and metabolic diseases in the offspring; however, how GDM along with maternal obesity interact to alter colonization remains unknown. We hypothesized that GDM individually and in combination with maternal overweight/obesity would alter gut microbial composition, diversity, and short-chain fatty acid (SCFA) levels in neonates. We investigated 46 full-term neonates born to normal-weight or overweight/obese mothers with and without GDM, accounting for confounders including cesarean delivery, lack of breastfeeding, and exposure to antibiotics. Gut microbiota in 2-week-old neonates born to mothers with GDM exhibited differences in abundance of 26 microbial taxa; 14 of which showed persistent differential abundance after adjusting for pre-pregnancy BMI. Key pioneering gut taxa, including potentially important taxa for establishing neonatal immunity, were reduced. *Lactobacillus*, *Flavonifractor*, *Erysipelotrichaceae*, and unspecified families in *Gammaproteobacteria* were significantly reduced in neonates from mothers with GDM. GDM was associated with an increase in microbes involved in suppressing early immune cell function (*Phascolarctobacterium*). No differences in infant stool SCFA levels by maternal phenotype were noted; however, significant correlations were found between microbial abundances and SCFA levels in neonates. Our results suggest that GDM alone and together with maternal overweight/obesity uniquely influences seeding of specific infant microbiota in patterns that set the stage for future risk of inflammatory and metabolic disease.

## Introduction

Compelling epidemiological and experimental data show a strong link between maternal metabolic health and the origins of obesity and metabolic disorders in subsequent generations (1). In particular, gestational diabetes mellitus (GDM) and maternal overweight/obesity (OW/OB) independently and together increase offspring risk for metabolic diseases associated with inflammation and weight gain, including obesity, type 2 diabetes, and non-alcoholic fatty liver disease (NAFLD) (1, 2). The development of several essential biological systems depends on proper infant gut microbe seeding and expansion; these systems include energy extraction from the diet, gut barrier function, and immune system maturation/education (3, 4). Maternal obesity has been shown to increase neonatal cytokine levels (5) and alter cytokine responsiveness of newborn innate immune cells in early life (6, 7), suggesting that maternal obesity influences childhood risk for metabolic disease, in part through dysregulation of the early immune system. Early colonization of the neonatal gut is a central component to the development of appropriate innate and adaptive immune responses through high levels of *Lactobacillus* (8, 9) and early priming by members of the phylum *Proteobacteria* (10). Multiple studies have shown that maternal obesity alters the composition of the infant gut microbiota throughout the first years of life (11–14); however, only a few data are available in infants born to mothers with GDM.

Recent studies reported that GDM was associated with unique changes in the gut microbiota composition in women during the first trimester of pregnancy (15, 16), third trimester (17–19), and postpartum (17) (as reviewed in (20)). Another study reported that women with GDM had a distinct gut microbiota composition in the second trimester (21), enough to provide discriminatory power to predict GDM status (22). However, no differences in gut microbiota were observed 5-years postpartum in women with previous GDM compared with women without a GDM diagnosis (23). Studies on the microbiota in neonates showed differences in microbe composition between infants born to mothers with and without GDM (24–27). These studies are often confounded by various perinatal conditions known to disrupt offspring microbiota colonization, such as mode of delivery, type of feeding, antibiotic usage, and maternal obesity or excess gestational weight gain (EWG). In addition, maternal diet (28, 29) and host genetics (30, 31) have been reported to impact the infant microbiota, although these are more difficult to determine and quantify in humans. Differences in breast milk composition might also contribute to the infant gut microbiota in breastfed infants (12, 32–34). Variability in the control of confounders in these studies raises caution when interpreting and comparing published data, while illustrating the need for further investigations in order to determine how to best characterize the gut microbiota in infants born to women with GDM.

Despite the explosion of data in the microbiome field, small, well-controlled, hypothesis directed studies have yet to identify the impact of GDM on the infant microbiome. Accordingly, we investigated whether GDM alone and in association with the common comorbidity obesity altered the microbiota colonization pattern and diversity in 2-week-old infants. As a secondary outcome, the influence of EWG alone and in association with OW/OB were also investigated. Short-chain fatty acids (SCFA), mainly acetate, butyrate, and propionate, in the infant gut have potent anti-inflammatory effects, and play an important role in shaping mechanisms underlying immune cell development (35); therefore, infant stool SCFA levels were assessed. In this study, we demonstrate significant decreases in early seeding of pioneer microbes in infants born to mothers with GDM that may play a role in future postnatal immune cell development, and associations between phyla and SCFAs in these infants.

## Materials and Methods

### Study Cohort

Subjects were enrolled in one of three ongoing longitudinal studies of mother-infant dyads between 2012 and 2017 in the Denver, Colorado metropolitan area. All aspects of the clinical studies were approved by the Colorado Multiple Institutional Review Board and the studies were registered at http://www.clinicaltrials.gov (NCT01693406, NCT02244814, and NCT00826904). All research was performed in accordance with relevant guidelines and regulations. Informed consent was obtained from all women and all planned to exclusively breastfeed for at least 4 months and were otherwise healthy. Women with any chronic medical diseases requiring treatment such as cardiopulmonary or renal disease, or pre-existing diabetes were excluded.

Women enrolled in this study were aged 20–39 years old. Maternal pre-pregnancy BMI was based on self-report of pre-pregnancy weight and measured height. Women were classified as normal weight (NW; <25 kg m^-2^) or overweight/obese (OW/OB; >28 kg m^−2^) based on pre-pregnancy BMI. GDM was diagnosed by Carpenter and Coustan criteria (36). Briefly, between gestation weeks 24–28, women who failed a non-fasting 1-h blood glucose measurement after 50 g oral glucose solution (≥135 mg dl^−1^) underwent a 3-h 100 g oral glucose tolerance test. Two or more abnormal blood glucose measurements at fasting, 1, 2, or 3 h per thresholds (≥95, 180, 155, or 140 mg dl^−1^) were considered positive for GDM. EWG was determined by the change in weight from self-reported pre-pregnancy weight to weight at 36–37 weeks gestation as measured by clinical staff at that study visit. EWG was determined using Institute of Medicine (IOM) standards which have recommended ranges of weight gain according to pre-pregnancy BMI. Women were deemed to have EWG if total weight gain was above IOM recommendations (16 and 9 kg for NW and OW/OB groups, respectively) or no EWG if weight gain was below these thresholds (37). All infants were born of a singleton pregnancy at term (>37 weeks gestation). Our inclusion criteria were vaginal delivery or cesarean delivery after trial of labor (ensuring exposure to the vaginal canal and associated microbiota), no antibiotics or probiotics except in the immediate peripartum period, and predominate breastfeeding in the first 2 weeks of life. Women with GDM were included only if managed by diet alone, without the use of medications (such as insulin or oral agents) to control blood glucose. Thus, we attempted to eliminate the major confounders that typically interfere with early infant microbial colonization of the gut.

Forty-six mother-infant pairs met the inclusion criteria. Infant adiposity (percent fat mass) was measured by air displacement plethysmography in a PEAPOD Infant Body Composition System (COSMED, Rome, Italy) at the 2-week postpartum visit. Of note, four infants were delivered *via* cesarean delivery after a trial of labor, four infants had formula supplement ranging from 2 to 8 ounces per week at 2 weeks of life, one mother had taken probiotics consistently for greater than 2 years, five women were given penicillin at delivery for Group B Streptococcus, and one mother received doxycycline antibiotics during the 2-week postpartum period.

### Microbiota Composition

At the 2-week postpartum visit, mothers brought an infant stool sample, collected within 24 h of visit and saved in a frozen diaper at home (~−20°C), which was then aliquoted and stored at −80°C. Profiling of stool bacteria was conducted by 16S rRNA gene sequencing, as outlined previously (12, 38). In brief, microbial DNA from all stool samples was extracted using the PowerFecal DNA Isolation kit (Qiagen, Germantown, MD, USA) per manufacturer’s instructions. Isolated DNA was quantified by quantitative PCR for targeted bacterial subgroups as previously (39, 40). Broad-range bacterial 16S rRNA gene amplicons were generated using barcoded primers 27F-YM (5′ - AGAGTTTGATYMTGGCTCAG) and 338R (5′ - TGCTGCCTCCCGTAGGAGT) targeting approximately 300 base pairs of the V1-V2 variable region (39, 41, 42). Primer 27F-YM was designed to enhance amplification of *Bifidobacterium* (41). Pooled amplicons were diluted to 15 pM, spiked with 25% of the Illumina PhiX control DNA, and paired-end sequenced on the Miseq platform with a 600-cycle v.3 reagent kit (Illumina, San Diego, CA, USA). Assembled sequences were aligned and classified with SINA (1.3.0-r23838) (43) using the 418,497 bacterial sequences in Silva 115NR99 (44) as reference configured to yield the Silva taxonomy. Closed-reference operational taxonomic units (OTUs) were produced by clustering sequences with identical taxonomic assignments. OTUs were analyzed at the genus level, the lowest taxonomic level generated by SINA. Stool microbiota profiling was successful in all samples. The median sequencing depth was 191682 with an IQR of 111622. The mean Good’s Coverage was 91.45% with a standard deviation of 2.6%.

### SCFA Collection and Measurement

Of the total cohort, we had 23 samples available for SCFA quantification (NW = 12 [GDM-NW = 5, No GDM-NW = 7; EWG-NW = 4, No EWG-NW = 8] and OW/OB = 11 [GDM-OW/OB = 6, No GDM-OW/OB = 5; EWG-OW/OB = 5, No EWG-OW/OB = 6]). Infant stool samples were weighed and diluted to equivalency with acidified water (pH 2.5) containing 1 mmol ethyl-butyric acid per liter as an internal standard. Samples were sonicated for 10 min, incubated at room temperature for 10 min, and then centrifuged at 10,000 × *g* for 10 min at room temperature. Supernatant was collected, re-centrifuged, and stored at −80°C until analysis, as previously (12, 45). Samples were analyzed using a 6890 series gas chromatograph with flame ionization detector (Agilent). Samples were injected at a 10:1 split ratio; the inlet was held at 228°C and the transfer line was held at 230°C. Separation of SCFAs was achieved on a 30m TG-WAX-A column (0.25-mm ID, 0.25-mm film thickness; Thermo Scientific) by using a temperature program as previously (45). Acetate, propionate, and butyrate were quantified using standards of commercially purchased compounds and samples were adjusted for extraction efficiency differences by normalizing to the internal standard.

### Statistical Analysis

All data manipulation, analyses, and graphics were conducted using R and RStudio (versions 3.5.1 and 1.1.456, respectively). The R package tidyMicro (version 1.48) was used for all analyses (46) and ggplot2 was used for visualizations (47). Maternal and infant characteristics were summarized with frequencies (%) for categorical variables and means (standard deviations) or median (interquartile range [IRQ]) for continuous variables. Differences in these characteristics were assessed by Fisher’s exact test for categorical variables and *t*-tests for continuous variables. Relative abundance (RA) was calculated as the number of sequencing reads of each taxon in a sample, standardized by the total number of sequences generated for that sample. Only taxa that were present in at least 5% of infants and had a RA of a least 0.1% in at least one infant were included in the analyses. Sequence counts for taxa that did not meet these requirements were aggregated into an “Other” category. These filtering requirements were applied at the phylum, family, and genus levels. Sequence counts that could not be classified to the taxonomic level of interest were left as unclassified counts of the lowest level possible. For example, during genus-level analyses, sequence reads only classifiable to a family level were analyzed as unclassified (i.e., unclassified *Lactobacillaceae*).

Alpha diversity measures Chao1, Shannon diversity (H = -sum(p_i_ *log2(p_i_)), where p_i_ is the RA of taxon i), and Shannon evenness ($\frac{H}{H_{\max}}$) were all calculated through 1,000 replicate re-samplings based on the genus-level OTU counts. The rarefaction level for alpha diversity measures was 57,228, the minimum sequencing depth of this cohort. UniFrac distances weighted by individual taxa counts and the Bray-Curtis dissimilarity were also calculated based on genus-level counts. Differences in infant alpha diversities were assessed using linear regression models and a *t*-test on the regression coefficients. Differences in infant UniFrac distances and beta diversities were assessed using a non-parametric permutation-based multivariate analysis of variation (PERMANOVA) test from the *vegan* package with 999 permutations. We tested for significant impacts from the co-occurrences between maternal GDM and OW/OB as well as maternal EWG and OW/OB for each diversity measure by including interaction terms between the two phenotypes in each model.

Differences in the RAs of phylum-, family-, and genus-level taxa were evaluated using generalized linear models (GLM) assuming a negative binomial distribution and log link function; the total number of sequences was used as an offset. Benjamini and Hochberg’s false discovery rate (FDR) correction was then applied to adjust for multiple comparisons within each taxon level with significance defined as FDR *p* value ≤0.05. For each taxon, a GLM model was first fit including an interaction term between maternal BMI and either GDM or EWG (separate models for GDM and EWG). If the interaction term was significant (FDR *p* < 0.05), it was determined that the effect of GDM (or EWG) on the taxa was modified by maternal BMI and results were reported for these models. If the interaction term was not significant, the interaction term was removed from the model and the independent effect of GDM (or EWG), after adjusting for OW/OB, was modeled and reported. Estimated relationships between taxa RA and maternal phenotype are reported as a rate ratio (RR), which was obtained by exponentiating the regression coefficients from the GLM models, and Wald confidence intervals were calculated for each RR. Stacked bar charts were constructed using estimated taxa RA obtained from the GLMs to visually display the estimated microbiota compositions by phenotypic group. The top 10 highest estimated overall abundance taxa were plotted while the remaining estimated taxa counts were aggregated into an “Other” category for readability.

Differences in butyrate, propionate, acetate, and total SCFAs were assessed using linear regression models and a *t*-test on the regression coefficients. For each SCFA, we tested for associations between the SCFA and the co-occurrence of maternal GDM and OW/OB as well as maternal EWG and OW/OB using interaction terms. Spearman correlations between SCFA measurements and the centered log ratio of genus-level counts were also calculated. A taxon with a correlation r≥|0.5| was considered to be clinically meaningful. These correlations were visualized using Rocky Mountain plots, a variation on the Manhattan plot; each line represents the strength of correlation for an individual taxon, with positive correlations extending up from the 0-correlation reference line and negative correlations extending down.

The three longitudinal studies in which samples were collected had limited preliminary data and minimal published data to generate sample size power calculations. Therefore, sample sizes were determined using estimates of microbiota variability and published standard deviations of desired measurements in NW women (12). Specifically, these parent studies were each powered to have 80% power to detect ~18% differences between phylum level abundances in the microbiota from infants of NW and OW/OB women with a Type I error of 0.05, requiring 20 infants per a group. Here, we mitigated Type I error rate inflation through an FDR correction for multiple comparisons. Importantly, the infants selected without exception were delivered vaginally or after a trial of labor, were predominantly breastfed, and with one exception, had no exposure to antibiotics outside of the peripartum period. Thus, we eliminated the major confounders that typically interfere with early infant microbial colonization.

## Results

### Cohort Characteristics

Forty-six women and their neonates met the inclusion criteria both at birth and at 2-weeks postpartum including vaginal delivery or trial of labor, breastfeeding as the main source of feeding, and no antibiotic exposure (except in the immediate peripartum period). We studied infants born to 13 women with GDM and 33 women without GDM (**Table 1**). Of these 46 women, 27 were NW (BMI = 22.5 ± 2.2 kg/m^2^) and 19 were OW/OB (BMI = 32.2 ± 3.9 kg/m^2^) (**Supplementary Table S1**). Fasting glucose levels at 37 weeks were not significantly different between women with and without GDM (78.0 ± 7.6 and 77.0 ± 7.1 mg/dl, respectively; *p* = 0.688; **Table 1**), indicating excellent fasting glycemic control in our cohort of GDM women. Adiposity of infants born to mothers with and without GDM was not different at 2 weeks of life. Infant adiposity was also not different between infants born to NW and OW/OB mothers (**Supplementary Table S1**) or to mothers with and without EWG (**Supplementary Table S2**). Overall, an even distribution of male and female infants was studied. Sample metadata with variables used in the analyses is shown in **Supplementary Table S3**.

**Table 1:** Maternal and infant characteristics stratified by maternal GDM.

	GDM	No GDM	*p* value
Maternal			
Weight group, *n* (%)			0.331
NW	6 (46.2)	21 (63.6)	
OW/OB	7 (53.8)	12 (36.4)	
Race/ethnicity, *n* (%)			0.089
Asian, non-Hispanic	4 (30.8)	1 (3.0)	
Black, Hispanic	0 (0)	1 (3.0)	
Black, non-Hispanic	1 (7.7)	2 (6.1)	
White, Hispanic	1 (7.7)	2 (6.1)	
White, non-Hispanic	7 (53.8)	27 (81.8)	
Age, y	32.1 (4.7)	31.5 (3.2)	0.614
Pre-pregnancy BMI, kg m^-2^	27.6 (5.5)	26.1 (5.8)	0.398
Gestational weight gain, kg	8.9 (4.7)	13.9 (6.3)	0.013
Primiparous, *n* (%)	4 (36.4)	16 (50.0)	0.285
Cesarean delivery*, *n* (%)	2 (15.4)	2 (6.1)	0.323
Fasting glucose, mg dl^−1^	78.0 (7.6)	77.0 (7.1)	0.688
Missing, *n* (%)	0 (0)	7 (21.2)	
Infant			
Gestational age, week	39.4 (0.9)	40.0 (1.0)	0.064
Birthweight, g	3.24 (0.36)	3.35 (0.51)	0.490
Fat mass, %	11.1 (3.3)	10.0 (4.0)	0.378
Missing, *n* (%)	1 (7.7)	2 (6.1)	
Sex, *n* (%)			1.000
Male	6 (46.2)	16 (48.5)	
Female	7 (53.8)	17 (51.5)	
Formula supplementation, *n* (%)	3 (23.1)	1 (3.0)	0.062

Data are expressed as count (%) or mean (SD).

*After trial of labor.

p values assessed by Fisher’s exact test or chi-square test for categorical variables and t-test for continuous variables.

### GDM Is Associated With Infant Microbiota Composition

**Figure 1:**
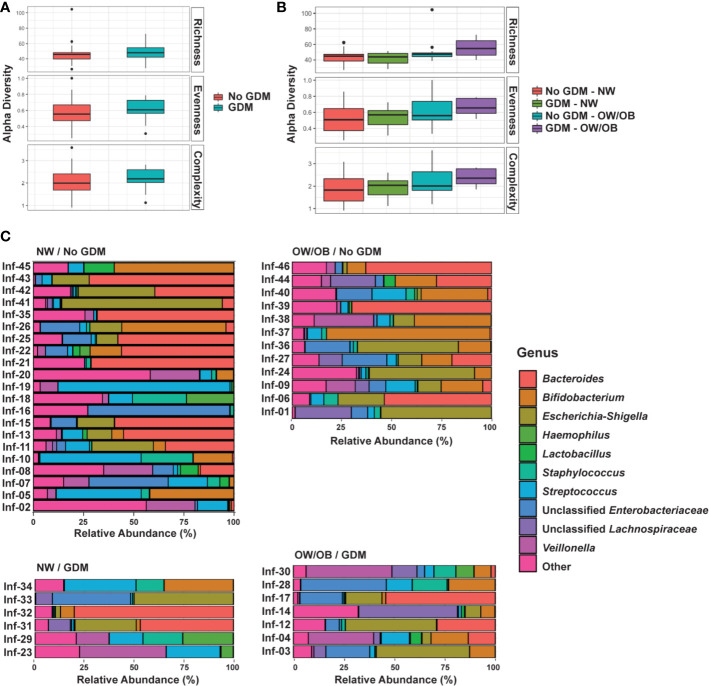
GDM and maternal overweight/obesity alter infant gut microbiota. Box plots of genus-level alpha diversities richness (Chao1), evenness (Shannon $\frac{H}{H_{\max}}$), and complexity (Shannon H) by **(A)** GDM and **(B)** GDM and weight group. Black dots represent outliers within strata. **(C)** Relative abundance of microbiota taxa for each infant at the genus level. Genus-level taxa shown are the 10 most abundant with the remaining classified as Other. Relative abundance estimated using negative binomial regression on 16S rRNA gene amplicon sequences from stool samples, shown as percent of total. GDM, gestational diabetes; NW, normal-weight; OW/OB, overweight/obese.

The associations between infant microbiota diversity and GDM status and OW/OB were examined to understand if GDM had an impact on the overall microbiota composition. No significant differences were observed in Shannon $\frac{H}{H_{\max}}$ or Shannon H alpha diversities (measures of genera evenness and complexity, respectively) with GDM either individually or together with OW/OB (**Figures 1A, B**, respectively; **Supplementary Table S4**). The association between the Chao1 measure of richness and maternal OW/OB in infants from mothers without GDM trended higher compared with NW mothers without GDM but did not reach statistical significance (*p* = 0.059). Maternal GDM, OW/OB, and the co-occurrence of these did not significantly impact Bray-Curtis dissimilarity or weighted UniFrac distances, both measures of beta diversity (**Supplementary Table S4**).

To understand the impact of GDM on individual microbes, we examined associations between the maternal phenotype and RA of individual taxa at the phylum, family, and genus levels. We first explored associations between GDM and RA modified by maternal BMI through interaction terms. If modification was not present (interaction FDR *p* < 0.05), we examined the associations between GDM and RA in additive models excluding the interaction term. After filtering low abundance taxa, we examined 7 phyla, 49 families, and 70 genera. The most abundant genera in the infant microbiota according to GDM status and maternal BMI is shown in **Figure 1C** and abundant phyla and families are shown in **Supplementary Figure 1**. The full data sets for family- and genus-level taxa are shown in **Supplementary Tables S5** and **S6**, respectively. Altogether, we identified 26 microbial taxa across these levels that were differentially abundant in the gut microbiota of infants from mothers with GDM.

**Figure 2:**
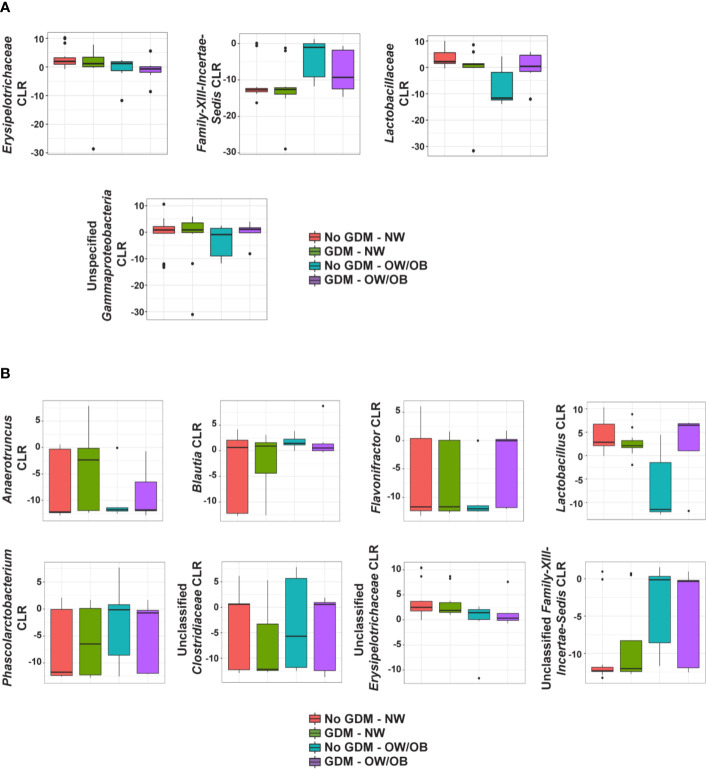
Summary of the impact of maternal GDM on infant gut microbial taxa in the interaction model. Box plots of centered log ratio (CLR) transformed **(A)** family- and **(B)** genus-level taxa counts by maternal GDM status and weight group. Taxa displayed are those whose relationship in infants from mothers with GDM differed significantly based on maternal weight group. GDM, gestational diabetes; NW, normal-weight; OW/OB, overweight/obese.

**Figure 3:**
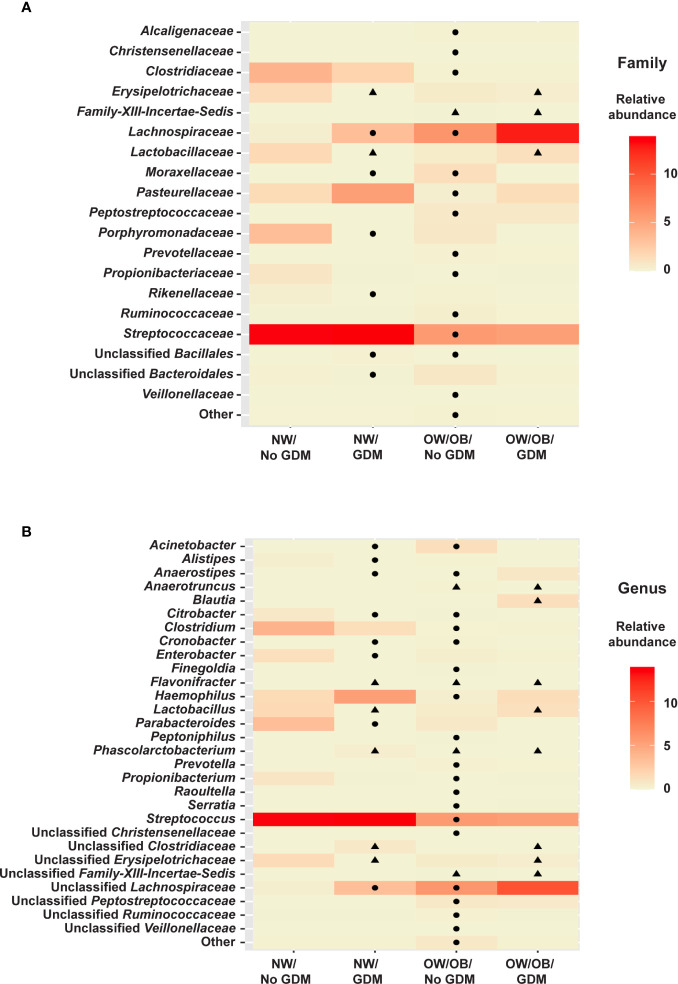
Heat maps of significant relationships between maternal phenotype and infant gut microbiota. Average taxa relative abundances are displayed by maternal phenotype with higher averages colored a deeper red. Cells with a shape inside indicate a significantly different (FDR adjusted *p* value <0.05) average relative abundance from the NW/No GDM group. Triangles indicate significant differences from the interaction models and circles indicate differences from the additive models for both **(A)** family- and **(B)** genus-level taxa.

No taxa at the phylum level were significantly different between infants born to mothers with and without GDM and modified by OW/OB (*p* > 0.05 for all interaction terms). Going further in the microbial taxonomy, the co-occurrence of GDM and maternal BMI were associated with taxa abundances at the family and genus levels as summarized in **Figure 2** and detailed in **Supplementary Tables S7** and **S8**, respectively. *Lactobacillaceae* (*p* < 0.001), *Erysipelotrichaceae* (*p* < 0.001), and unspecified families in the class *Gammaproteobacteria* (*p* = 0.005) showed decreased abundance with the co-occurrence of GDM and OW/OB, and this co-occurrence was associated with increased Family-XIII-*Incertae-Sedis* (*p* = 0.014) (**Figure 3A**, **Supplementary Table S7**). Associations with significant alterations in microbe abundances were also observed at the genus level for the co-occurrence of GDM and maternal OW/OB (**Figure 3B**, **Supplementary Table S8**). Notably, *Lactobacillus* was decreased (*p* < 0.001) and *Phascolarctobacterium* was increased (*p* < 0.001) in the co-occurrence of these maternal phenotypes. The presence of GDM in NW mothers was associated with RAs of taxa at the family level: decreased *Lactobacillaceae* (*p* < 0.001), *Erysipelotrichaceae* (*p* < 0.001), and unspecified families in *Gammaproteobacteria* (*p* < 0.001) (**Figure 3A**, **Supplementary Table S7**). Associations with significant alterations in microbe abundances and GDM were also observed at the genus level including decreased *Lactobacillus* (*p* < 0.001) and *Flavonifractor* (*p* = 0.003) and increased *Phascolarctobacterium* (*p* < 0.001) (**Figure 3B**, **Supplementary Table S8**).

**Figure 4:**
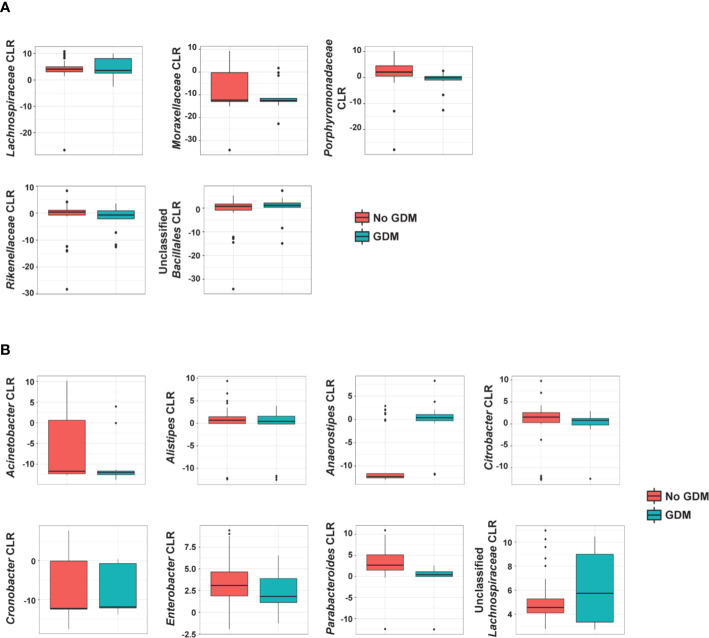
Summary of the impact of maternal GDM on infant gut microbial taxa in the additive model. Box plots of centered log ratio (CLR) transformed **(A)** family- and **(B)** genus-level taxa counts by maternal GDM status. Taxa displayed are those whose abundance changed significantly in infants from mothers with GDM after controlling for maternal weight group. GDM, gestational diabetes.

In the additive models, 14 taxa were significantly different in the offspring of mothers with GDM compared to those without, after adjusting for pre-pregnancy BMI (summarized in **Figure 4**). At the phylum level, GDM was associated with decreased *Fusobacteria* (*p* = 0.002) (**Supplementary Table S9**) after controlling for OW/OB. The presence of GDM alone was associated with a number of abundance differences at the family level (**Figure 3A**, **Supplementary Table S10**). GDM after controlling for OW/OB was associated with decreased *Porphyromonadaceae* (*p* < 0.001), *Rikenellaceae* (*p* < 0.001), and *Moraxellaceae* (*p* < 0.001) and an increase in *Lachnospiraceae* (*p* = 0.005). Four genera within *Gammaproteobacteria* were significantly decreased in GDM (**Figure 3B**, **Supplementary Table S11**).

### Maternal OW/OB Is Associated With Infant Microbiota Composition After Controlling for GDM

The co-occurrence and additive models also allowed for the investigation of the impact of maternal BMI on infant microbiota in the presence of GDM. The presence of OW/OB in the interaction models comparing the microbiota in infants born to NW mothers without GDM showed a significant decrease in unspecified families in *Gammaproteobacteria* (*p* = 0.004) and an increase in Family-XIII-*Incertae-Sedis* (*p* < 0.001) (**Figure 3A**, **Supplementary Table S7**). Notably, for genus-level taxa, *Phascolarctobacterium* showed increased abundance (*p* = 0.005) (**Figure 3B**, **Supplementary Table S8**). In the additive model, maternal OW/OB alone after controlling for GDM was associated with increased *Fusobacteria* (*p* = 0.002) (**Supplementary Table S9**). Family- and genus-level taxa with associations with maternal OW/OB are shown in **Supplementary Tables S10** and **S11**, respectively.

### Effects of EWG on Infant Microbiota Diversity and Composition

Since EWG beyond recommended limits is common in OW/OB mothers (48) and has been suggested to modify the infant gut microbiota (49, 50), we investigated the effects of maternal EWG, OW/OB, and the interaction of these phenotypes on infant gut microbiota diversity. Increases in Chao1, Shannon $\frac{H}{H_{\max}}$, and Shannon H were all significantly associated with maternal OW/OB after accounting for EWG (*p* = 0.002, *p* = 0.031, *p* = 0.031 respectively; **Supplementary Figure S2A**, **Supplementary Table S12**), but not EWG itself. Similarly, the Bray-Curtis dissimilarity index was altered in infants based on maternal OW/OB (*p* = 0.043), but EWG neither altered beta diversity nor changed the impact of maternal OW/OB (**Supplementary Table S12**). Maternal EWG, OW/OB, and the co-occurrence of these did not significantly impact infants’ weighted UniFrac distances (**Supplementary Table S12**).

To investigate the impact of EWG on the RA of individual microbes, we followed the same approach as with GDM by first exploring the co-occurrence of EWG and maternal BMI through interactions, and, if not significant, fitting additive models with the two phenotypes. Taxa with the highest estimated RAs of gut microbes in offspring by maternal EWG and OW/OB are displayed at the phylum, family, and genus levels (**Supplementary Figures S2B, S3A, B**, respectively) and the full family- and genus-level data sets are shown in **Supplementary Tables S13** and **S14**, respectively. Overall, we identified 26 taxa that had different RAs of microbes from infants of mothers with EWG (**Supplementary Table S15**). EWG, OW/OB, and the co-occurrence of these conditions analyzed in the interaction model were associated with significant alterations in RAs at the phylum and family levels (**Supplementary Figure S4A** and **Supplementary Tables S16** and **S17**, respectively). Maternal OW/OB and EWG groups showed significant or trending associations with increased abundance of the phylum *Fusobacteria* (*p* < 0.001 and *p* = 0.086, respectively), yet the presence of both OW/OB and EWG was associated with significantly decreased *Fusobacteria* abundance (*p* < 0.001). At the family level, a significantly higher abundance of *Corynebacteriaceae* was associated with the co-occurrence of both EWG and OW/OB (*p* < 0.001) as well as EWG (*p* < 0.001) and OW/OB (*p* = 0.025) alone. Genus-level taxa with significant associations with maternal EWG, OW/OB, and their co-occurrence are shown in **Supplementary Figure S4B** and **Supplementary Table S18**.

Taxa that were not significantly altered by the co-occurrence between EWG and OW/OB were re-analyzed in the additive model. No significant associations were observed at the phylum level; however, at the family level, both EWG and OW/OB groups were independently associated with RA alterations after controlling for one another (**Supplementary Figure S4A**, **Supplementary Table S19**). Notably, EWG was associated with decreased abundance of *Prevotellaceae* (*p* < 0.001), *Rikenellaceae* (*p* < 0.001), and *Veillonellaceae* (*p* = 0.011) and increased *Porphyromonadaceae* (*p* = 0.010) and *Alcaligenaceae* (*p* = 0.006). Maternal OW/OB was associated with increased *Prevotellaceae* (*p* < 0.001), *Lachnospiraceae* (*p* < 0.001), and *Ruminococcaceae* (*p* = 0.006) and decreased *Propionibacteriaceae* (*p* = 0.018) and *Pasteurellaceae* (*p* = 0.019). Genus-level taxa with significant independent associations with either EWG or OW/OB are shown in **Supplementary Figure S4B** and **Supplementary Table S20**. Of note, maternal OW/OB was associated with an increase in *Finegoldia* (*p* = 0.031) and *Peptoniphilus* (*p* < 0.001) in the phylum *Firmicutes*.

### SCFA Levels in Infants Correlate With Gut Microbes

**Figure 5:**
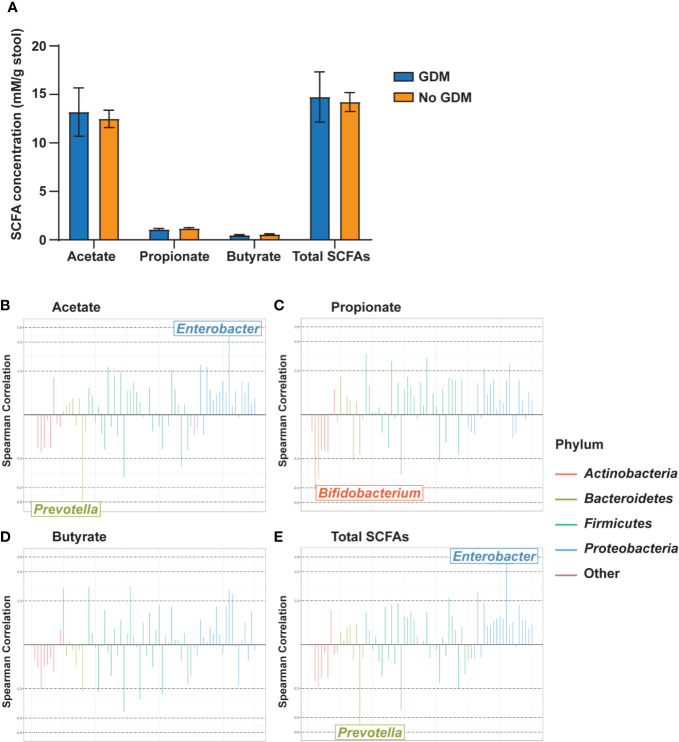
Gut microbiota genus-level taxa correlate with infant stool SCFA levels. **(A)** Infant stool SCFA levels by maternal GDM status. Rocky Mountain Plots representing **(B)** acetate, **(C)** propionate, **(D)** butyrate, and **(E)** total SCFA measurements with a center log ratio transformation of genus-level counts. Correlations were derived using Spearman Correlation with *r* > |0.5| indicating a meaningful correlation. *n* = 23 for each measure.

In our cohort, stool collected from infants at 2 weeks of age was dominated by acetate levels followed by propionate and then butyrate at a ratio of approximately 25:2:1 (**Figure 5A**). Maternal phenotypes and their co-occurrences were not associated with alterations in levels of infant stool SCFAs (**Supplementary Tables S21** and **S22**). However, several taxa were correlated with significant changes in SCFA levels. Acetate levels were positively correlated with the RA of *Enterobacter* in the *Gammaproteobacteria* and negatively correlated with *Prevotella* (**Figure 5B**). Propionate was negatively correlated with *Bifidobacterium* (**Figure 5C**). Butyrate did not meaningfully correlate with any taxa (**Figure 5D**). Total SCFAs correlated with the same microbial families as acetate (**Figure 5E**), likely due to the predominance of this SCFA in the infant gut. All SCFA measurements had consistent, though weak, negative correlations with taxa from the phylum *Actinobacteria* and positive correlations with taxa from the phylum *Proteobacteria* (**Supplementary Table S23**).

## Discussion

In this study, we examined if GDM alone and together with maternal obesity during pregnancy drive early offspring gut microbial colonization in a carefully selected cohort of full-term, predominantly breastfed, vaginally-exposed infants, without pre- or postnatal antibiotic exposure. We observed differences in abundance of 26 microbial taxa in stool from neonates born to mothers with GDM; 14 of which showed persistent differential abundance after adjusting for pre-pregnancy BMI. Given that obesity is a major driver of insulin resistance, our results suggest that exposures in infants born to mothers with GDM other than insulin resistance contributes to altered offspring microbiota colonization. Whether a unique maternal gut microbiota, breast milk microbiota, or both contributes to infant microbiota colonization, and whether factors in the infant gut make the environment more suitable to disorder/dysbiosis in offspring from mothers with GDM are unknown. Because infants with dysbiotic microbiota are prone to fluctuations in microbial colonization early in life, changes noted at 2 weeks of age may become less dominant over time. In the future, it will also be important to carry out longitudinal studies in these infants to 3 years of life when the microbiota becomes more stable.

A number of key pioneering gut taxa, including *Lactobacillus*, *Flavonifractor*, *Lactobacillaceae*, *Rikenellaceae*, *Erysipelotrichaceae*, and unspecified families in *Gammaproteobacteria*, were decreased in 2-week-old infants from mothers with GDM. Early microbial contact is involved in the initiation and perpetuation of pivotal immune activation and responsiveness that is central to the development of both innate and adaptive immunity (51–53). Various studies have established a correlation between factors that disrupt the gut microbiota during pregnancy and breastfeeding and immune and metabolic disorders later in life (54, 55). For example, microbiota composition shifts were found to occur in infants that may predict the development of type 1 diabetes (56). Vatanen *et al*. demonstrated that *Bacteroides* species in microbiota of infants with high susceptibility to allergies and type 1 diabetes produced a lipopolysaccharide (LPS) subtype that inhibited immunostimulatory activity of *Escherichia coli* LPS *in vitro* (10). Intraperitoneal injection of *E. coli*-derived LPS led to endotoxin tolerance in immune cells and delayed onset of type 1 diabetes in a mouse model, whereas *Bacteroides* LPS was not protective (10). We found reduced *Citrobacter* in offspring from mothers with GDM; this genus is closely related to other taxa in *Enterobacteriaceae*, including *E. coli*, with similar LPS modifications (57). Decreased *Citrobacter* RA suggests a potential for reduced inflammatory or immunomodulatory roles to the host response to endotoxin tolerance or innate training (58). It remains to be seen whether specific genera or a combination of species imparts different effects on the development of immunity in the infant, as there is enormous diversity and interplay within members of these families. In this regard, however, we recently showed that microbiota from 2-week-old infants born to obese mothers increased obesity and NAFLD in gnotobiotic mice, due in part to increased gut permeability and the inability of bone-marrow derived macrophages to engulf bacteria and protect against inflammation (38, 59). This illustrates that alterations in gut microbiota have functional consequences through the programming of fundamental pathways involved in innate immunity and obesity prior to disease.

Simultaneous exposure to GDM and OW/OB was associated with a decrease in *Lactobacillaceae*, potentially contributing negatively to early immunological development as this taxon was previously shown to contribute to innate immune system education in early life (60). This is consistent with data showing that the genus *Lactobacillus* is reduced in meconium from infants born to mothers with GDM (24). In addition, infants exposed to GDM and OW/OB independently and simultaneous GDM and OW/OB exposure had increased *Phascolarctobacterium.* This genus was previously shown to be elevated in women with GDM compared with pregnant women with normal glucose status (18, 22), suggesting it is vertically transmitted from mother to infant. Interestingly, *Phascolarctobacterium* has been shown to influence intestinal inflammation through its role as a succinate consumer (61). Infants born to mothers with GDM showed a significant association with elevated *Lachnospiraceae* after controlling for OW/OB. *Lachnospiraceae* is enriched in women with GDM (15, 17, 18) and in adults with type 2 diabetes (62, 63), suggesting that it is a marker associated with impaired glycemic control transmitted to infants of GDM mothers.

Additive models of taxa RA demonstrated a prominent role for OW/OB in independently shaping offspring microbiota. OW/OB alone was associated with shifts in microbes, previously reported in infants of OW/OB mothers (11), including decreased *Pasteurellaceae* and increased *Finegoldia* and *Peptoniphilus* after controlling for either GDM or EWG. The increased *Lachnospiraceae* and *Ruminococcaceae* associated with OW/OB (after controlling for either GDM or EWG) have previously been positively correlated with leptin levels (both families), maternal BMI (*Lachnospiraceae*), and insulin levels (*Ruminococcaceae*) in pregnant obese women (64), implying maternal transfer to the infant during delivery and/or lactation. After controlling for either GDM or EWG, infants of OW/OB mothers also have decreased *Streptococcaceae*, which is consistent with our previous study of 2-week-old infants of obese mothers (12). Analysis of EWG (after controlling for OW/OB) found an association with increased *Porphyromonadaceae*; a family previously associated with hepatic steatosis and inflammation in inflammasome-deficient mice (65) as well as in human NASH (66).

No differences in SCFAs by maternal phenotype were noted in our study. However, we did find significant correlations between microbial RAs and SCFA levels among predominantly/exclusively breastfed neonates. For example, acetate levels were positively correlated with the RA of *Enterobacter* in *Gammaproteobacteria*. The formation of acetate by *E. coli*, a pioneering aerobe in *Gammaproteobacteria*, is a commonly observed phenomenon (67, 68); however, RA of *Gammaproteobacteria* was reduced in OW/OB and GDM infants, suggesting multiple sources for acetate. Acetate can also be consumed by many rapidly proliferating gut microbes, possibly leading to changes in microbial community composition, which in turn could influence host metabolism.

In our study, we used the V1-V2 region for sequencing the 16S rRNA genome. It is important to recognize that these short reads (using one to three variable regions) are unable to accurately and confidently discriminate species-level taxonomic classification (69) and may have underestimated the relative levels of *Bifidobacterium* in the infants (70). We acknowledge that analysis of microbial communities at the species level could provide a very different perspective to the one afforded by genus-level abundance estimates. Our results are limited to offspring from mothers whose GDM was controlled by diet. The role of insulin and metformin on the maternal gut microbiome and SCFA levels in mothers and infants remains to be studied. We did not measure the breast milk microbiome, and this deserves further study.

Overall, many of our findings in the microbiota of infants from mothers with GDM mirror those in maternal GDM gut dysbiosis (17, 21) and those found in infants with compromised immunological function (9, 71). Regarding possible mechanisms for infant prenatal colonization in mothers with GDM, speculation suggests that the intrauterine maternal microbiota is a source of first colonizers in the infant gut, but the existence of an *in utero* microbiota remains controversial. A recent study suggests that microbial products or metabolites are detected in the human fetal intestine and drive *in utero* immune development and education (72); however, the source of the bacteria is unknown. Gestation-only colonization of mice with *E. coli* was reported to modify the intestinal mucosal innate immune system and transcriptome of the offspring (73). Similarly, in a prospective cohort of 26 mother-infant dyads, a high-fat maternal gestational diet was associated with distinct variations in the neonatal gut microbial composition (meconium), which persisted to 4 to 6 weeks of age (74), suggesting a maternal diet-driven microbiota during development. Given differences in early colonizers in 2-week-old offspring of mothers with GDM, it is not unlikely that the neonatal microbiome and immune system may be programmed by maternal factors at different stages of development depending on the cell type and location (75). This may include changes in the maternal microbiome, human milk oligosaccharides, SCFAs or inflammation in mothers with GDM (17, 19, 76).

In conclusion, these results demonstrate an independent role of maternal GDM in the infant gut microbial composition. Additional research in large, prospective, human birth cohorts (both maternal and infant) will be necessary to understand how these early microbiota changes may interact with host components to drive aspects of infant immune development and disease pathways over time in offspring from mothers with GDM. Small changes may become less dominant over time; however, early patterns of microbial succession over the first year of life have been correlated with susceptibility to immune diseases later in life (51–53). This represents a significant challenge but a unique opportunity to discover pathways to developmental programming in the neonate.

## Data Availability Statement

16S rRNA gene sequences and associate metadata generated and analyzed during the current study have been deposited in the NCBI Sequence Read Archive and are available through BioProject ID: PRJNA558340. https://www.ncbi.nlm.nih.gov/sra.

## Ethics Statement

The studies involving human participants were reviewed and approved by Colorado Multiple Institutional Review Board, University of Colorado Anschutz Medical Campus, Aurora, CO, United States. The patients/participants provided their written informed consent to participate in this study.

## Author Contributions

TS, RJ, and JF wrote the manuscript. TS, TW, and DI performed the experiments. CC, CR, and MK performed bioinformatic and biostatistical analysis of the data. RJ formatted the manuscript, tables, and figures. BY, NK, TH, and LB designed and conducted the clinical studies from which samples were generated. JF and DF provided guidance on the study design, data interpretation, and analysis. All authors contributed to the article and approved the submitted version.

## Funding

This study was supported by the American Diabetes Association/Glaxo Smith Kline Targeted Research Award (1-13-GSK-13, to JF), the Thrasher Research Fund Early Career Award (to BY), the University of Colorado GI and Liver Innate Immune Program (to DF), the NIH National Institute of Diabetes and Digestive and Kidney Diseases (F32 HD0978068 to BY; R01 DK078645 to LB; R01 DK101659 to TH and LB).

## Conflict of Interest

JF is a consultant to the scientific advisory board of Janssen Pharmaceuticals.

The remaining authors declare that the research was conducted in the absence of any commercial or financial relationships that could be construed as a potential conflict of interest.
