# Supplementary material for: Gestational Diabetes Is Uniquely Associated With Altered Early Seeding of the Infant Gut Microbiota
Source: Front Endocrinol (Lausanne). 2020 Nov 27;11:603021. doi: 10.3389/fendo.2020.603021 (PMC7729132; doi:10.3389/fendo.2020.603021)
Supplement: Supplementary file 1 [file DataSheet_1.pdf]

Supplementary Materials for:

"Gestational diabetes is uniquely associated with altered early seeding of the infant gut microbiota"

Taylor K. Soderborg[1,†], Charles M. Carpenter[2,†], Rachel C. Janssen[1,&], Tiffany L. Weir[3], Charles E. Robertson[4], Diana Ir[4], Bridget E. Young[5,#], Nancy F. Krebs[5], Teri L. Hernandez[6,7], Linda A. Barbour[6,8], Daniel N. Frank[4], Miranda Kroehl[2] & Jacob E. Friedman[1,6,&]*

[1]Department of Pediatrics, Section of Neonatology, University of Colorado Anschutz Medical Campus, Aurora, CO, United States

[2]Division of Biostatistics and Epidemiology, University of Colorado School of Public Health, University of Colorado Anschutz Medical Campus, Aurora, CO, United States

[3]Department of Food Science and Human Nutrition, Colorado State University, Fort Collins, CO, United States

[4]Department of Medicine, Division of Infectious Disease, University of Colorado Anschutz Medical Campus, Aurora, CO, United States

[5]Department of Pediatrics, Section of Nutrition, University of Colorado Anschutz Medical Campus, Aurora, CO, United States

[6]Department of Medicine, Division of Endocrinology, Metabolism & Diabetes, University of Colorado Anschutz Medical Campus, Aurora, CO, United States

[7]College of Nursing, University of Colorado Anschutz Medical Campus, Aurora, CO, United States

[8]Department of Obstetrics and Gynecology, Division of Maternal Fetal Medicine, University of Colorado Anschutz Medical Campus, Aurora, CO, United States.

[†]These authors contributed equally to this work and share first authorship.

* Correspondence:

Jacob E Friedman

jed-friedman@ouhsc.edu

[&]Present address: Harold Hamm Diabetes Center, University of Oklahoma Health Sciences Center, Oklahoma City, OK, United States.

[#]Present address: Department of Pediatrics; Allergy and Immunology, University of Rochester School of Medicine and Dentistry, Rochester, NY, United States.

Supplementary Table S1 Maternal and infant characteristics stratified by maternal weight group

	NW (*n* = 27)	OW/OB (*n* = 19)	*p* value
Maternal			
GDM, *n* (%)			0.331
GDM	6 (22.2)	7 (36.8)	
No GDM	21 (77.8)	12 (63.2)	
EWG, *n* (%)			0.373
EWG	9 (33.3)	9 (47.4)	
No EWG	18 (66.7)	10 (52.6)	
Race/ethnicity, *n* (%)			0.520
Asian, non-Hispanic	4 (14.8)	1 (5.3)	
Black, Hispanic	0 (0)	1 (5.3)	
Black, non-Hispanic	1 (3.7)	2 (10.5)	
White, Hispanic	2 (7.4)	1 (5.3)	
White, non-Hispanic	20 (74.1)	14 (73.7)	
Age, y	31.4 (3.4)	31.9 (4.1)	0.617
Pre-pregnancy BMI, kg m^{-2}	22.5 (2.2)	32.2 (3.9)	< 0.001
Gestational weight gain, kg	14.9 (4.7)	9.0 (6.6)	0.001
Primiparous, *n* (%)	11 (42.3)	7 (41.2)	0.457
Cesarean delivery*, *n* (%)	3 (11.1)	1 (5.3)	0.499
Fasting glucose, mg dL^{-1}	75.7 (5.8)	79.6 (8.5)	0.098
Missing, *n* (%)	4 (14.8)	3 (15.8)	
Infant			
Gestational age, week	39.9 (0.8)	39.6 (1.1)	0.279
Birthweight, kg	3.29 (0.44)	3.37 (0.52)	0.561
Fat mass, %	10.3 (3.7)	10.2 (4.0)	0.934
Missing, *n* (%)	2 (7.4)	1 (5.3)	
Sex, *n* (%)			0.245
Male	15 (55.6)	7 (36.8)	
Female	12 (44.4)	12 (63.2)	
Formula supplementation, *n* (%)	2 (7.0)	2 (10.5)	0.719

Data are expressed as count (%) or mean (SD).
*After trial of labor.
p values assessed by Fisher's exact test for categorical variables, and *t*-test for continuous variables.

Supplementary Table S2 Maternal and infant characteristics stratified by maternal EWG

	EWG *n* = 18	No EWG *n* = 28	*p* value
Maternal			
Weight group, *n* (%)			0.373
NW	9 (50.0)	18 (64.3)	
OW/OB	9 (50.0)	10 (35.7)	

Race/ethnicity, *n* (%)			0.266
Asian, non-Hispanic	0 (0)	5 (17.9)	
Black, Hispanic	1 (5.6)	0 (0)	
Black, non-Hispanic	1 (5.6)	2 (7.1)	
White, Hispanic	1 (5.6)	2 (7.1)	
White, non-Hispanic	15 (83.3)	19 (67.9)	
Age, y	32.2 (3.1)	31.3 (4.0)	0.405
Pre-pregnancy BMI, kg m^{-2}	27.1 (5.3)	26.1 (6.0)	0.568
Gestational weight gain, kg	17.2 (5.0)	9.4 (5.0)	<0.001
Primiparous, *n* (%)	9 (50.0)	11 (44.0)	0.485
Cesarean delivery*, *n* (%)	3 (16.7)	1 (3.6)	0.130
Fasting glucose, mg dL^{-1}	77.3 (6.5)	77.4 (7.7)	0.976
Missing, *n* (%)	4 (22.2)	3 (10.7)	
Infant			
Gestational age, week	39.9 (1.1)	39.8 (0.9)	0.791
Birthweight, g	3.36 (0.55)	3.30 (0.42)	0.687
Fat mass, %	9.4 (4.0)	10.8 (3.6)	0.233
Missing, *n* (%)	1 (5.6)	2 (7.1)	
Sex, *n* (%)			1.000
Male	9 (50.0)	13 (46.4)	
Female	9 (50.0)	15 (53.6)	
Formula supplementation, *n* (%)	1 (5.6)	3 (10.7)	0.555

Data are expressed as count (%) or mean (SD).
*After trial of labor.
p values assessed by Fisher's exact test for categorical variables and *t*-test for continuous variables.

Supplementary Table S3 Subject metadata

Subject ID	NW/OB	GDM	EWG
INF1	OW/OB	No GDM	No EWG
INF2	NW	No GDM	No EWG
INF3	OW/OB	GDM	No EWG
INF4	OW/OB	GDM	No EWG
INF5	NW	No GDM	EWG
INF6	OW/OB	No GDM	EWG
INF7	NW	No GDM	No EWG
INF8	NW	No GDM	EWG
INF9	OW/OB	No GDM	No EWG
INF10	NW	No GDM	No EWG
INF11	NW	No GDM	No EWG
INF12	OW/OB	GDM	EWG
INF13	NW	No GDM	No EWG
INF14	OW/OB	GDM	No EWG
INF15	NW	No GDM	No EWG
INF16	NW	No GDM	No EWG
INF17	OW/OB	GDM	No EWG
INF18	NW	No GDM	EWG
INF19	NW	No GDM	No EWG

INF20	NW	No GDM	EWG
INF21	NW	No GDM	No EWG
INF22	NW	No GDM	EWG
INF23	NW	GDM	No EWG
INF24	OW/OB	No GDM	No EWG
INF25	NW	No GDM	No EWG
INF26	NW	No GDM	EWG
INF27	OW/OB	No GDM	EWG
INF28	OW/OB	GDM	No EWG
INF29	NW	GDM	No EWG
INF30	OW/OB	GDM	EWG
INF31	NW	GDM	No EWG
INF32	NW	GDM	No EWG
INF33	NW	GDM	No EWG
INF34	NW	GDM	No EWG
INF35	NW	No GDM	EWG
INF36	OW/OB	No GDM	EWG
INF37	OW/OB	No GDM	EWG
INF38	OW/OB	No GDM	No EWG
INF39	OW/OB	No GDM	EWG
INF40	OW/OB	No GDM	EWG
INF41	NW	No GDM	EWG
INF42	NW	No GDM	No EWG
INF43	NW	No GDM	No EWG
INF44	OW/OB	No GDM	EWG
INF45	NW	No GDM	EWG
INF46	OW/OB	No GDM	No EWG

Supplementary Table S4 Alpha and beta diversities by GDM and maternal overweight/obesity

	Group	Estimate difference in means	95% CI	*p* value
Alpha diversity				
Chao1	GDM	-1.4286	(-12.66,9.8)	0.799
Chao1	OW/OB	8.4365	(-0.34,17.22)	0.059
Chao1	GDM:OW/OB	5.3975	(-10.71,21.5)	0.503
Shannon $\frac{H}{H_{max}}$	GDM	0.0053	(-0.15,0.16)	0.944
Shannon $\frac{H}{H_{max}}$	OW/OB	0.0897	(-0.03,0.21)	0.134
Shannon $\frac{H}{H_{max}}$	GDM:OW/OB	0.0421	(-0.18,0.26)	0.697
Shannon H	GDM	0.0188	(-0.53,0.56)	0.945
Shannon H	OW/OB	0.3229	(-0.1,0.75)	0.134
Shannon H	GDM:OW/OB	0.1517	(-0.63,0.93)	0.697
Beta diversity				
Bray-Curtis	GDM	--	--	0.666
Bray-Curtis	OW/OB	--	--	0.052
Bray-Curtis	GDM:OW/OB	--	--	0.995
UniFrac				
Weighted	GDM	--	--	0.662
Weighted	OW/OB	--	--	0.232

Weighted	GDM:OW/OB	--	--	0.992

p values assessed by *t*-test with 42 degrees of freedom for alpha diversity.
p value assessed by PERMANOVA with 999 permutations for beta diversity and UniFrac.

Supplementary Table S5 Family-level taxa average relative abundances by maternal GDM status and BMI

Taxa	GDM	BMI group	RA
Actinomycetaceae	No GDM	NW	0.2973 (0.68)
Actinomycetaceae	No GDM	OW/OB	0.0677 (0.2)
Actinomycetaceae	GDM	NW	0.045 (0.11)
Actinomycetaceae	GDM	OW/OB	0.2296 (0.51)
Alcaligenaceae	No GDM	NW	0.0257 (0.11)
Alcaligenaceae	No GDM	OW/OB	0.1532 (0.53)
Alcaligenaceae	GDM	NW	0.001 (0)
Alcaligenaceae	GDM	OW/OB	0.1291 (0.34)
Bacillaceae	No GDM	NW	9e-04 (0)
Bacillaceae	No GDM	OW/OB	0.3802 (1.32)
Bacillaceae	GDM	NW	0 (0)
Bacillaceae	GDM	OW/OB	8e-04 (0)
Bacteroidaceae	No GDM	NW	25.8909 (29.16)
Bacteroidaceae	No GDM	OW/OB	20.1659 (27.11)
Bacteroidaceae	GDM	NW	21.2363 (34.48)
Bacteroidaceae	GDM	OW/OB	14.1182 (20.66)
Bifidobacteriaceae	No GDM	NW	10.1584 (18.17)
Bifidobacteriaceae	No GDM	OW/OB	20.4027 (23.07)
Bifidobacteriaceae	GDM	NW	7.305 (13.76)
Bifidobacteriaceae	GDM	OW/OB	10.2291 (8.4)
Christensenellaceae	No GDM	NW	0.0016 (0)
Christensenellaceae	No GDM	OW/OB	0.0212 (0.07)
Christensenellaceae	GDM	NW	0.0025 (0)
Christensenellaceae	GDM	OW/OB	0.0025 (0)
Clostridiaceae	No GDM	NW	4.0595 (8.76)
Clostridiaceae	No GDM	OW/OB	0.128 (0.29)
Clostridiaceae	GDM	NW	1.9803 (3.2)
Clostridiaceae	GDM	OW/OB	0.1573 (0.21)
Coriobacteriaceae	No GDM	NW	0.4659 (0.98)
Coriobacteriaceae	No GDM	OW/OB	1.1242 (2.7)
Coriobacteriaceae	GDM	NW	0.4811 (1.14)
Coriobacteriaceae	GDM	OW/OB	0.5518 (0.51)
Corynebacteriaceae	No GDM	NW	0.1003 (0.38)
Corynebacteriaceae	No GDM	OW/OB	0.0332 (0.04)
Corynebacteriaceae	GDM	NW	0.0162 (0.02)
Corynebacteriaceae	GDM	OW/OB	0.0353 (0.04)

Desulfovibrionaceae	No GDM	NW	0.019 (0.09)
Desulfovibrionaceae	No GDM	OW/OB	0.4492 (1.55)
Desulfovibrionaceae	GDM	NW	0.156 (0.24)
Desulfovibrionaceae	GDM	OW/OB	0.0013 (0)
Enterobacteriaceae	No GDM	NW	21.6288 (24.14)
Enterobacteriaceae	No GDM	OW/OB	26.4492 (25.14)
Enterobacteriaceae	GDM	NW	22.8655 (34.85)
Enterobacteriaceae	GDM	OW/OB	31.4759 (25.67)
Enterococcaceae	No GDM	NW	0.1393 (0.51)
Enterococcaceae	No GDM	OW/OB	0.427 (0.9)
Enterococcaceae	GDM	NW	0.2211 (0.54)
Enterococcaceae	GDM	OW/OB	0.2958 (0.69)
Erysipelotrichaceae	No GDM	NW	1.4693 (4.22)
Erysipelotrichaceae	No GDM	OW/OB	0.5515 (1.1)
Erysipelotrichaceae	GDM	NW	0.0043 (0)
Erysipelotrichaceae	GDM	OW/OB	0.4038 (1.06)
Family-XIII-Incertae-Sedis	No GDM	NW	1e-04 (0)
Family-XIII-Incertae-Sedis	No GDM	OW/OB	0.0583 (0.2)
Family-XIII-Incertae-Sedis	GDM	NW	8e-04 (0)
Family-XIII-Incertae-Sedis	GDM	OW/OB	0.0013 (0)
Lachnospiraceae	No GDM	NW	0.2934 (0.67)
Lachnospiraceae	No GDM	OW/OB	5.977 (9.82)
Lachnospiraceae	GDM	NW	3.314 (5.07)
Lachnospiraceae	GDM	OW/OB	12.6992 (24.45)
Lactobacillaceae	No GDM	NW	1.6512 (3.78)
Lactobacillaceae	No GDM	OW/OB	0.4903 (1.59)
Lactobacillaceae	GDM	NW	0.0073 (0.01)
Lactobacillaceae	GDM	OW/OB	1.2038 (2.01)
Micrococcaceae	No GDM	NW	0.2495 (0.83)
Micrococcaceae	No GDM	OW/OB	0.0895 (0.24)
Micrococcaceae	GDM	NW	0.0214 (0.03)
Micrococcaceae	GDM	OW/OB	0.083 (0.18)
Moraxellaceae	No GDM	NW	6e-04 (0)
Moraxellaceae	No GDM	OW/OB	1.2814 (4.15)
Moraxellaceae	GDM	NW	0 (0)
Moraxellaceae	GDM	OW/OB	0.0083 (0.02)
Negativicoccus	No GDM	NW	0 (0)
Negativicoccus	No GDM	OW/OB	0.0772 (0.26)
Negativicoccus	GDM	NW	1e-04 (0)
Negativicoccus	GDM	OW/OB	0.0011 (0)
Neisseriaceae	No GDM	NW	0.0095 (0.03)
Neisseriaceae	No GDM	OW/OB	0.024 (0.04)

Neisseriaceae	GDM	NW	0.0037 (0.01)
Neisseriaceae	GDM	OW/OB	0.0604 (0.16)
Pasteurellaceae	No GDM	NW	1.5081 (5.19)
Pasteurellaceae	No GDM	OW/OB	0.2985 (0.45)
Pasteurellaceae	GDM	NW	5.288 (10.15)
Pasteurellaceae	GDM	OW/OB	1.4066 (3.34)
Peptostreptococcaceae	No GDM	NW	0.0081 (0.02)
Peptostreptococcaceae	No GDM	OW/OB	0.7128 (2.33)
Peptostreptococcaceae	GDM	NW	0.0037 (0)
Peptostreptococcaceae	GDM	OW/OB	0.5927 (1.56)
Porphyromonadaceae	No GDM	NW	3.3017 (9.5)
Porphyromonadaceae	No GDM	OW/OB	0.7565 (1.35)
Porphyromonadaceae	GDM	NW	0.0046 (0.01)
Porphyromonadaceae	GDM	OW/OB	0.0251 (0.04)
Prevotellaceae	No GDM	NW	0.0015 (0)
Prevotellaceae	No GDM	OW/OB	0.2115 (0.72)
Prevotellaceae	GDM	NW	0.029 (0.06)
Prevotellaceae	GDM	OW/OB	0.1103 (0.23)
Propionibacteriaceae	No GDM	NW	0.8502 (3.16)
Propionibacteriaceae	No GDM	OW/OB	0.0307 (0.07)
Propionibacteriaceae	GDM	NW	0.053 (0.12)
Propionibacteriaceae	GDM	OW/OB	0.0761 (0.14)
Rikenellaceae	No GDM	NW	0.3404 (1.49)
Rikenellaceae	No GDM	OW/OB	0.1515 (0.4)
Rikenellaceae	GDM	NW	0.006 (0.01)
Rikenellaceae	GDM	OW/OB	0.0073 (0.01)
Ruminococcaceae	No GDM	NW	0.0745 (0.17)
Ruminococcaceae	No GDM	OW/OB	0.3478 (0.62)
Ruminococcaceae	GDM	NW	0.0783 (0.1)
Ruminococcaceae	GDM	OW/OB	0.1079 (0.22)
Staphylococcaceae	No GDM	NW	3.686 (7.68)
Staphylococcaceae	No GDM	OW/OB	2.0394 (2.04)
Staphylococcaceae	GDM	NW	6.0702 (8.7)
Staphylococcaceae	GDM	OW/OB	4.4623 (6.89)
Streptococcaceae	No GDM	NW	13.4823 (21.26)
Streptococcaceae	No GDM	OW/OB	5.6711 (5.34)
Streptococcaceae	GDM	NW	13.8872 (15.46)
Streptococcaceae	GDM	OW/OB	5.3372 (5.63)
Unclassified *Bacillales*	No GDM	NW	0.0135 (0.03)
Unclassified *Bacillales*	No GDM	OW/OB	0.005 (0.01)
Unclassified *Bacillales*	GDM	NW	0.2583 (0.59)
Unclassified *Bacillales*	GDM	OW/OB	0.0269 (0.03)

Unclassified *Bacteroidales*	No GDM	NW	0.3459 (1.01)
Unclassified *Bacteroidales*	No GDM	OW/OB	1.5429 (3.81)
Unclassified *Bacteroidales*	GDM	NW	1.0532 (2.41)
Unclassified *Bacteroidales*	GDM	OW/OB	2.019 (4.12)
Unclassified *Clostridiales*	No GDM	NW	2.1389 (6.38)
Unclassified *Clostridiales*	No GDM	OW/OB	1.6425 (3.37)
Unclassified *Clostridiales*	GDM	NW	3.9923 (6.34)
Unclassified *Clostridiales*	GDM	OW/OB	0.9986 (1.33)
Unclassified *Lactobacillales*	No GDM	NW	0.0936 (0.31)
Unclassified *Lactobacillales*	No GDM	OW/OB	0.0097 (0.01)
Unclassified *Lactobacillales*	GDM	NW	0.0054 (0.01)
Unclassified *Lactobacillales*	GDM	OW/OB	0.0175 (0.03)
Unclassified *Selenomonadales*	No GDM	NW	0 (0)
Unclassified *Selenomonadales*	No GDM	OW/OB	1e-04 (0)
Unclassified *Selenomonadales*	GDM	NW	0.0249 (0.06)
Unclassified *Selenomonadales*	GDM	OW/OB	1e-04 (0)
Veillonellaceae	No GDM	NW	5e-04 (0)
Veillonellaceae	No GDM	OW/OB	0.0313 (0.11)
Veillonellaceae	GDM	NW	9e-04 (0)
Veillonellaceae	GDM	OW/OB	0.0016 (0)
Xanthomonadaceae	No GDM	NW	0.0206 (0.09)
Xanthomonadaceae	No GDM	OW/OB	0.0454 (0.16)
Xanthomonadaceae	GDM	NW	0 (0)
Xanthomonadaceae	GDM	OW/OB	0 (0)
Other	No GDM	NW	0.0184 (0.02)
Other	No GDM	OW/OB	0.095 (0.26)
Other	GDM	NW	0.0252 (0.05)
Other	GDM	OW/OB	0.0553 (0.05)

Data are expressed as mean (SD).

Supplementary Table S6 Genus-level taxa average relative abundances by maternal GDM status and BMI

Taxa	GDM	BMI group	RA
Acinetobacter	No GDM	NW	5e-04 (0)
Acinetobacter	No GDM	OW/OB	1.2814 (4.15)
Acinetobacter	GDM	NW	0 (0)
Acinetobacter	GDM	OW/OB	0.0078 (0.02)
Actinomyces	No GDM	NW	0.2955 (0.67)
Actinomyces	No GDM	OW/OB	0.0656 (0.2)
Actinomyces	GDM	NW	0.0449 (0.11)
Actinomyces	GDM	OW/OB	0.229 (0.51)
Alistipes	No GDM	NW	0.3401 (1.49)

Alistipes	No GDM	OW/OB	0.1512 (0.4)
Alistipes	GDM	NW	0.006 (0.01)
Alistipes	GDM	OW/OB	0.0064 (0.01)
Anaerostipes	No GDM	NW	0.0015 (0.01)
Anaerostipes	No GDM	OW/OB	0.0234 (0.08)
Anaerostipes	GDM	NW	0.0056 (0.01)
Anaerostipes	GDM	OW/OB	0.8011 (2.12)
Anaerotruncus	No GDM	NW	2e-04 (0)
Anaerotruncus	No GDM	OW/OB	0.1277 (0.44)
Anaerotruncus	GDM	NW	1e-04 (0)
Anaerotruncus	GDM	OW/OB	3e-04 (0)
Atopobium	No GDM	NW	0.1261 (0.54)
Atopobium	No GDM	OW/OB	0.0125 (0.02)
Atopobium	GDM	NW	0 (0)
Atopobium	GDM	OW/OB	0.0707 (0.19)
Bacteroides	No GDM	NW	25.8909 (29.16)
Bacteroides	No GDM	OW/OB	20.1659 (27.11)
Bacteroides	GDM	NW	21.2363 (34.48)
Bacteroides	GDM	OW/OB	14.1182 (20.66)
Bifidobacterium	No GDM	NW	10.1584 (18.17)
Bifidobacterium	No GDM	OW/OB	20.4027 (23.07)
Bifidobacterium	GDM	NW	7.305 (13.76)
Bifidobacterium	GDM	OW/OB	10.2191 (8.39)
Bilophila	No GDM	NW	0.0189 (0.09)
Bilophila	No GDM	OW/OB	0.2891 (1)
Bilophila	GDM	NW	0.1556 (0.24)
Bilophila	GDM	OW/OB	0.0013 (0)
Blautia	No GDM	NW	0.007 (0.02)
Blautia	No GDM	OW/OB	0.0083 (0.01)
Blautia	GDM	NW	0.0076 (0.01)
Blautia	GDM	OW/OB	1.2624 (3.33)
Butyricimonas	No GDM	NW	0.0248 (0.11)
Butyricimonas	No GDM	OW/OB	3e-04 (0)
Butyricimonas	GDM	NW	0 (0)
Butyricimonas	GDM	OW/OB	0 (0)
Citrobacter	No GDM	NW	0.6081 (2.75)
Citrobacter	No GDM	OW/OB	0.0616 (0.2)
Citrobacter	GDM	NW	0.0023 (0)
Citrobacter	GDM	OW/OB	0.0028 (0)
Clostridium	No GDM	NW	4.0443 (8.76)
Clostridium	No GDM	OW/OB	0.1167 (0.29)
Clostridium	GDM	NW	1.2841 (2.27)

Clostridium	GDM	OW/OB	0.1554 (0.21)
Collinsella	No GDM	NW	0.1381 (0.62)
Collinsella	No GDM	OW/OB	0.7397 (2.56)
Collinsella	GDM	NW	0.3508 (0.86)
Collinsella	GDM	OW/OB	0.1749 (0.44)
Corynebacterium	No GDM	NW	0.0971 (0.38)
Corynebacterium	No GDM	OW/OB	0.0209 (0.01)
Corynebacterium	GDM	NW	0.0162 (0.02)
Corynebacterium	GDM	OW/OB	0.021 (0.03)
Cronobacter	No GDM	NW	4e-04 (0)
Cronobacter	No GDM	OW/OB	0.125 (0.43)
Cronobacter	GDM	NW	1e-04 (0)
Cronobacter	GDM	OW/OB	4e-04 (0)
Desulfovibrio	No GDM	NW	1e-04 (0)
Desulfovibrio	No GDM	OW/OB	0.1601 (0.55)
Desulfovibrio	GDM	NW	3e-04 (0)
Desulfovibrio	GDM	OW/OB	0 (0)
Dorea	No GDM	NW	3e-04 (0)
Dorea	No GDM	OW/OB	2e-04 (0)
Dorea	GDM	NW	0.0011 (0)
Dorea	GDM	OW/OB	0.5283 (1.4)
Eggerthella	No GDM	NW	0.0216 (0.1)
Eggerthella	No GDM	OW/OB	0.0993 (0.27)
Eggerthella	GDM	NW	0.0012 (0)
Eggerthella	GDM	OW/OB	0.0744 (0.1)
Enterobacter	No GDM	NW	1.204 (3.23)
Enterobacter	No GDM	OW/OB	0.3507 (0.81)
Enterobacter	GDM	NW	0.0208 (0.04)
Enterobacter	GDM	OW/OB	0.1383 (0.26)
Enterococcus	No GDM	NW	0.1393 (0.51)
Enterococcus	No GDM	OW/OB	0.427 (0.9)
Enterococcus	GDM	NW	0.2211 (0.54)
Enterococcus	GDM	OW/OB	0.2958 (0.69)
Escherichia-Shigella	No GDM	NW	10.7222 (19.45)
Escherichia-Shigella	No GDM	OW/OB	18.3626 (21.99)
Escherichia-Shigella	GDM	NW	13.938 (21.47)
Escherichia-Shigella	GDM	OW/OB	17.4008 (20.27)
Faecalibacterium	No GDM	NW	0.0033 (0.01)
Faecalibacterium	No GDM	OW/OB	0.0059 (0.01)
Faecalibacterium	GDM	NW	0.0134 (0.03)
Faecalibacterium	GDM	OW/OB	0.021 (0.05)
Finegoldia	No GDM	NW	0.0035 (0.01)

Finegoldia	No GDM	OW/OB	0.0681 (0.2)
Finegoldia	GDM	NW	4e-04 (0)
Finegoldia	GDM	OW/OB	0.0123 (0.02)
Flavonifractor	No GDM	NW	0.0237 (0.07)
Flavonifractor	No GDM	OW/OB	0.001 (0)
Flavonifractor	GDM	NW	1e-04 (0)
Flavonifractor	GDM	OW/OB	0.0023 (0)
Gemella	No GDM	NW	0.0339 (0.05)
Gemella	No GDM	OW/OB	0.0598 (0.11)
Gemella	GDM	NW	0.0391 (0.04)
Gemella	GDM	OW/OB	0.0861 (0.14)
Haemophilus	No GDM	NW	1.5071 (5.19)
Haemophilus	No GDM	OW/OB	0.2971 (0.45)
Haemophilus	GDM	NW	5.288 (10.15)
Haemophilus	GDM	OW/OB	1.4066 (3.34)
Klebsiella	No GDM	NW	0.0038 (0.01)
Klebsiella	No GDM	OW/OB	5e-04 (0)
Klebsiella	GDM	NW	0 (0)
Klebsiella	GDM	OW/OB	0.0181 (0.05)
Lactobacillus	No GDM	NW	1.6512 (3.78)
Lactobacillus	No GDM	OW/OB	0.4903 (1.59)
Lactobacillus	GDM	NW	0.0073 (0.01)
Lactobacillus	GDM	OW/OB	1.2038 (2.01)
Negativicoccus	No GDM	NW	0 (0)
Negativicoccus	No GDM	OW/OB	0.0772 (0.26)
Negativicoccus	GDM	NW	1e-04 (0)
Negativicoccus	GDM	OW/OB	0.0011 (0)
Neisseria	No GDM	NW	0.0094 (0.03)
Neisseria	No GDM	OW/OB	0.0211 (0.04)
Neisseria	GDM	NW	0.0032 (0)
Neisseria	GDM	OW/OB	0.0603 (0.16)
Pantoea	No GDM	NW	0.0455 (0.21)
Pantoea	No GDM	OW/OB	0 (0)
Pantoea	GDM	NW	2.1619 (5.3)
Pantoea	GDM	OW/OB	0 (0)
Parabacteroides	No GDM	NW	3.2762 (9.46)
Parabacteroides	No GDM	OW/OB	0.622 (1.25)
Parabacteroides	GDM	NW	0.004 (0.01)
Parabacteroides	GDM	OW/OB	0.0094 (0.02)
Peptoniphilus	No GDM	NW	1e-04 (0)
Peptoniphilus	No GDM	OW/OB	0.0514 (0.16)
Peptoniphilus	GDM	NW	0.001 (0)

Peptoniphilus	GDM	OW/OB	0.0172 (0.03)
Phascolarctobacterium	No GDM	NW	5e-04 (0)
Phascolarctobacterium	No GDM	OW/OB	0.0088 (0.03)
Phascolarctobacterium	GDM	NW	0.3843 (0.94)
Phascolarctobacterium	GDM	OW/OB	0.0018 (0)
Porphyromonas	No GDM	NW	0 (0)
Porphyromonas	No GDM	OW/OB	0.1325 (0.46)
Porphyromonas	GDM	NW	0 (0)
Porphyromonas	GDM	OW/OB	0.0149 (0.04)
Prevotella	No GDM	NW	0.0014 (0)
Prevotella	No GDM	OW/OB	0.2112 (0.72)
Prevotella	GDM	NW	0.0288 (0.06)
Prevotella	GDM	OW/OB	0.1093 (0.23)
Propionibacterium	No GDM	NW	0.8502 (3.16)
Propionibacterium	No GDM	OW/OB	0.0301 (0.07)
Propionibacterium	GDM	NW	0.053 (0.12)
Propionibacterium	GDM	OW/OB	0.0761 (0.14)
Raoultella	No GDM	NW	0.0222 (0.1)
Raoultella	No GDM	OW/OB	2e-04 (0)
Raoultella	GDM	NW	2e-04 (0)
Raoultella	GDM	OW/OB	1e-04 (0)
Rothia	No GDM	NW	0.2493 (0.83)
Rothia	No GDM	OW/OB	0.0886 (0.24)
Rothia	GDM	NW	0.0214 (0.03)
Rothia	GDM	OW/OB	0.083 (0.18)
Serratia	No GDM	NW	0 (0)
Serratia	No GDM	OW/OB	0.001 (0)
Serratia	GDM	NW	0 (0)
Serratia	GDM	OW/OB	0.0515 (0.14)
Staphylococcus	No GDM	NW	3.686 (7.68)
Staphylococcus	No GDM	OW/OB	2.0394 (2.04)
Staphylococcus	GDM	NW	6.0702 (8.7)
Staphylococcus	GDM	OW/OB	4.4623 (6.89)
Stenotrophomonas	No GDM	NW	0.0206 (0.09)
Stenotrophomonas	No GDM	OW/OB	0.0454 (0.16)
Stenotrophomonas	GDM	NW	0 (0)
Stenotrophomonas	GDM	OW/OB	0 (0)
Streptococcus	No GDM	NW	13.4335 (21.25)
Streptococcus	No GDM	OW/OB	5.6598 (5.34)
Streptococcus	GDM	NW	13.8717 (15.45)
Streptococcus	GDM	OW/OB	5.3255 (5.62)
Sutterella	No GDM	NW	0.0253 (0.11)

Sutterella	No GDM	OW/OB	0.1526 (0.53)
Sutterella	GDM	NW	7e-04 (0)
Sutterella	GDM	OW/OB	0.1289 (0.34)
Unclassified *Christensenellaceae*	No GDM	NW	0.0016 (0)
Unclassified *Christensenellaceae*	No GDM	OW/OB	0.0212 (0.07)
Unclassified *Christensenellaceae*	GDM	NW	0.0025 (0)
Unclassified *Christensenellaceae*	GDM	OW/OB	0.0025 (0)
Unclassified *Clostridiaceae*	No GDM	NW	0.0152 (0.05)
Unclassified *Clostridiaceae*	No GDM	OW/OB	0.0113 (0.04)
Unclassified *Clostridiaceae*	GDM	NW	0.6963 (1.09)
Unclassified *Clostridiaceae*	GDM	OW/OB	0.0019 (0)
Unclassified *Coriobacteriaceae*	No GDM	NW	0.1487 (0.41)
Unclassified *Coriobacteriaceae*	No GDM	OW/OB	0.2725 (0.88)
Unclassified *Coriobacteriaceae*	GDM	NW	0.123 (0.28)
Unclassified *Coriobacteriaceae*	GDM	OW/OB	0.2188 (0.37)
Unclassified *Enterobacteriaceae*	No GDM	NW	9.0224 (17.11)
Unclassified *Enterobacteriaceae*	No GDM	OW/OB	7.5459 (8.59)
Unclassified *Enterobacteriaceae*	GDM	NW	6.7416 (16.02)
Unclassified *Enterobacteriaceae*	GDM	OW/OB	13.8633 (15.53)
Unclassified *Erysipelotrichaceae*	No GDM	NW	1.4688 (4.22)
Unclassified *Erysipelotrichaceae*	No GDM	OW/OB	0.5502 (1.1)
Unclassified *Erysipelotrichaceae*	GDM	NW	0.0043 (0)
Unclassified *Erysipelotrichaceae*	GDM	OW/OB	0.4015 (1.05)
Unclassified *Family-XIII-Incertae-Sedis*	No GDM	NW	1e-04 (0)
Unclassified *Family-XIII-Incertae-Sedis*	No GDM	OW/OB	0.0583 (0.2)
Unclassified *Family-XIII-Incertae-Sedis*	GDM	NW	8e-04 (0)
Unclassified *Family-XIII-Incertae-Sedis*	GDM	OW/OB	0.0013 (0)
Unclassified *Lachnospiraceae*	No GDM	NW	0.2802 (0.67)
Unclassified *Lachnospiraceae*	No GDM	OW/OB	5.9382 (9.82)
Unclassified *Lachnospiraceae*	GDM	NW	3.2832 (5.07)
Unclassified *Lachnospiraceae*	GDM	OW/OB	10.0897 (17.73)
Unclassified *Peptostreptococcaceae*	No GDM	NW	0.0081 (0.02)
Unclassified *Peptostreptococcaceae*	No GDM	OW/OB	0.7128 (2.33)
Unclassified *Peptostreptococcaceae*	GDM	NW	0.0037 (0)
Unclassified *Peptostreptococcaceae*	GDM	OW/OB	0.5908 (1.56)
Unclassified *Ruminococcaceae*	No GDM	NW	0.0442 (0.13)
Unclassified *Ruminococcaceae*	No GDM	OW/OB	0.209 (0.5)
Unclassified *Ruminococcaceae*	GDM	NW	0.0535 (0.08)
Unclassified *Ruminococcaceae*	GDM	OW/OB	0.076 (0.16)
Unclassified *Streptococcaceae*	No GDM	NW	0.0481 (0.16)
Unclassified *Streptococcaceae*	No GDM	OW/OB	0.0111 (0.02)
Unclassified *Streptococcaceae*	GDM	NW	0.0155 (0.03)

Unclassified *Streptococcaceae*	GDM	OW/OB	0.0114 (0.02)
Unclassified *Veillonellaceae*	No GDM	NW	5e-04 (0)
Unclassified *Veillonellaceae*	No GDM	OW/OB	0.0313 (0.11)
Unclassified *Veillonellaceae*	GDM	NW	9e-04 (0)
Unclassified *Veillonellaceae*	GDM	OW/OB	0.0016 (0)
Veillonella	No GDM	NW	5.6455 (8.46)
Veillonella	No GDM	OW/OB	4.7846 (8.94)
Veillonella	GDM	NW	10.0544 (17.76)
Veillonella	GDM	OW/OB	11.1034 (18.33)
Other	No GDM	NW	0.0674 (0.15)
Other	No GDM	OW/OB	0.5128 (1.62)
Other	GDM	NW	0.0612 (0.09)
Other	GDM	OW/OB	0.1288 (0.12)

Data expressed as mean (SD).

Supplementary Table S7 Family-level taxa negative binomial models with significant GDM:OW/OB interactions

Taxa	Group	RR	95% CI	Z	FDR *p* value
Lactobacillaceae	GDM	0.00	(5e-04, 0.0357)	-5.09	< 0.0001
Lactobacillaceae	OW/OB	0.30	(0.0587, 1.501)	-1.47	0.2474
Lactobacillaceae	GDM:OW/OB	0.73	(0.1033, 5.1458)	4.15	0.0001
Family-XIII-Incertae-Sedis	GDM	6.69	(0.3625, 123.3632)	1.28	0.3339
Family-XIII-Incertae-Sedis	OW/OB	484.50	(50.1776, 4678.2232)	5.34	< 0.0001
Family-XIII-Incertae-Sedis	GDM:OW/OB	10.89	(0.7118, 166.4754)	-2.76	0.0141
Erysipelotrichaceae	GDM	0.00	(4e-04, 0.0231)	-5.53	< 0.0001
Erysipelotrichaceae	OW/OB	0.37	(0.0759, 1.8488)	-1.21	0.3609
Erysipelotrichaceae	GDM:OW/OB	0.27	(0.0399, 1.8745)	3.67	7e-04
Unspecified Families in *Gammaproteobacteria*	GDM	0.00	(5e-04, 0.0315)	-5.25	< 0.0001
Unspecified Families in *Gammaproteobacteria*	OW/OB	0.08	(0.0156, 0.373)	-3.18	0.004
Unspecified Families in *Gammaproteobacteria*	GDM:OW/OB	0.03	(0.0045, 0.2083)	3.08	0.0052

Rate ratios (RR) compared with NW/No GDM infant group from exponentiated beta coefficients or sums of beta coefficients for interaction term (GDM:OW/OB). Wald confidence intervals for rate ratios. Z-score and FDR *p* value for original (non-exponentiated) beta coefficients.

Supplementary Table S8 Genus-level taxa negative binomial models with significant GDM:OW/OB interactions

Taxa	Group	RR	95% CI	Z	FDR *p* value
Lactobacillus	GDM	0.00	(5e-04, 0.0357)	-5.09	0.0000
Lactobacillus	OW/OB	0.30	(0.0587, 1.501)	-1.47	0.2382
Lactobacillus	GDM:OW/OB	0.73	(0.1033, 5.1458)	4.15	0.0001
Blautia	GDM	1.10	(0.1846, 6.5151)	0.10	0.9442

Blautia	OW/OB	1.18	(0.2944, 4.7554)	0.24	0.8865
Blautia	GDM:OW/OB	182.67	(34.5918, 964.6228)	3.81	0.0004
Anaerotruncus	GDM	0.50	(0.0182, 13.6187)	-0.41	0.8270
Anaerotruncus	OW/OB	745.06	(105.0424, 5284.6414)	6.62	0.0000
Anaerotruncus	GDM:OW/OB	1.90	(0.1461, 24.7797)	-2.44	0.0322
Flavonifractor	GDM	0.01	(3e-04, 0.1194)	-3.31	0.0025
Flavonifractor	OW/OB	0.04	(0.0058, 0.3104)	-3.11	0.0048
Flavonifractor	GDM:OW/OB	0.10	(0.0092, 1.0584)	2.93	0.0080
Phascolarctobacterium	GDM	733.39	(79.0751, 6801.9295)	5.81	0.0000
Phascolarctobacterium	OW/OB	16.68	(2.8463, 97.7181)	3.12	0.0047
Phascolarctobacterium	GDM:OW/OB	3.49	(0.4035, 30.1537)	-5.00	0.0000
Unclassified *Clostridiaceae*	GDM	45.88	(3.6267, 580.4618)	2.96	0.0075
Unclassified *Clostridiaceae*	OW/OB	0.75	(0.1023, 5.4652)	-0.29	0.8746
Unclassified *Clostridiaceae*	GDM:OW/OB	0.12	(0.0108, 1.4208)	-3.00	0.0066
Unclassified *Erysipelotrichaceae*	GDM	0.00	(4e-04, 0.0231)	-5.53	0.0000
Unclassified *Erysipelotrichaceae*	OW/OB	0.37	(0.0759, 1.8488)	-1.21	0.3534
Unclassified *Erysipelotrichaceae*	GDM:OW/OB	0.27	(0.0399, 1.8745)	3.67	0.0007
Unclassified *Family-XIII-Incertae-Sedis*	GDM	6.69	(0.3625, 123.3632)	1.28	0.3249
Unclassified *Family-XIII-Incertae-Sedis*	OW/OB	484.50	(50.1776, 4678.2232)	5.34	0.0000
Unclassified *Family-XIII-Incertae-Sedis*	GDM:OW/OB	10.89	(0.7118, 166.4754)	-2.76	0.0136

Rate ratios (RR) compared with NW/No GDM infant group from exponentiated beta coefficients or sums of beta coefficients for interaction term (GDM:OW/OB). Wald confidence intervals for rate ratios. Z-score and FDR *p* value for original (non-exponentiated) beta coefficients.

Supplementary Table S9 Phylum-level taxa negative binomial models with significant GDM or OW/OB main effects, controlling for other factor

Taxa	Group	RR	95% CI	Z	FDR *p* value
Fusobacteria	GDM	0.02	(0.0015, 0.1738)	-3.40	0.0016
Fusobacteria	OW/OB	28.55	(4.0913, 199.2139)	3.38	0.0016

Rate ratios (RR) compared with NW/No GDM infant group from exponentiated beta coefficients. Wald confidence intervals for rate ratios. Z-score and FDR *p* value for original (non-exponentiated) beta coefficients.

Supplementary Table S10 Family-level taxa negative binomial models with significant GDM or OW/OB main effects, controlling for other factor

Taxa	Group	RR	95% CI	Z	FDR *p* value
Propionibacteriaceae	GDM	0.77	(0.1269, 4.6871)	-0.28	0.8486
Propionibacteriaceae	OW/OB	0.08	(0.0158, 0.4289)	-2.97	0.0066
Porphyromonadaceae	GDM	0.01	(0.0026, 0.048)	-6.03	0.0000
Porphyromonadaceae	OW/OB	0.49	(0.1292, 1.8624)	-1.05	0.4232
Prevotellaceae	GDM	6.58	(1.1054, 39.1391)	2.07	0.0724
Prevotellaceae	OW/OB	64.19	(12.5342, 328.7398)	4.99	0.0000
Rikenellaceae	GDM	0.03	(0.0064, 0.1438)	-4.41	0.0000

Rikenellaceae	OW/OB	0.59	(0.1443, 2.4483)	-0.72	0.5876
Streptococcaceae	GDM	0.99	(0.4514, 2.1546)	-0.03	0.9722
Streptococcaceae	OW/OB	0.41	(0.2004, 0.8368)	-2.45	0.0293
Christensenellaceae	GDM	0.64	(0.158, 2.5783)	-0.63	0.6422
Christensenellaceae	OW/OB	7.06	(1.9929, 24.9941)	3.03	0.0055
Clostridiaceae	GDM	0.80	(0.2005, 3.1809)	-0.32	0.8274
Clostridiaceae	OW/OB	0.04	(0.0117, 0.1463)	-4.94	0.0000
Lachnospiraceae	GDM	5.80	(1.8889, 17.7926)	3.07	0.0049
Lachnospiraceae	OW/OB	12.90	(4.6249, 35.9617)	4.89	0.0000
Peptostreptococcaceae	GDM	0.63	(0.1786, 2.2153)	-0.72	0.5876
Peptostreptococcaceae	OW/OB	105.22	(33.408, 331.4043)	7.95	0.0000
Ruminococcaceae	GDM	0.63	(0.21, 1.8705)	-0.84	0.5364
Ruminococcaceae	OW/OB	3.31	(1.2166, 8.9806)	2.34	0.0366
Veillonellaceae	GDM	0.57	(0.0467, 7.0071)	-0.44	0.7481
Veillonellaceae	OW/OB	27.89	(2.8844, 269.7357)	2.88	0.0086
Alcaligenaceae	GDM	0.29	(0.0304, 2.7901)	-1.07	0.4202
Alcaligenaceae	OW/OB	12.55	(1.5913, 98.8985)	2.40	0.0327
Pasteurellaceae	GDM	4.07	(1.0027, 16.4907)	1.96	0.0898
Pasteurellaceae	OW/OB	0.22	(0.0601, 0.7777)	-2.34	0.0366
Moraxellaceae	GDM	0.00	(0.0034, 0.0054)	-46.85	0.0000
Moraxellaceae	OW/OB	3018.53	(2069.7638, 4402.2098)	41.62	0.0000
Unclassified *Bacillales*	GDM	11.24	(3.9801, 31.7145)	4.57	0.0000
Unclassified *Bacillales*	OW/OB	0.26	(0.0999, 0.6779)	-2.76	0.0120

Rate ratios (RR) compared with NW/No GDM infant group from exponentiated beta coefficients. Wald confidence intervals for rate ratios. Z-score and FDR *p* value for original (non-exponentiated) beta coefficients.

Supplementary Table S11 Genus-level taxa negative binomial models with significant GDM or OW/OB main effects, controlling for other factor

Taxa	Group	RR	95% CI	Z	FDR *p* value
Propionibacterium	GDM	0.78	(0.1288, 4.7752)	-0.26	0.8622
Propionibacterium	OW/OB	0.08	(0.0155, 0.4225)	-2.98	0.0063
Parabacteroides	GDM	0.01	(0.0014, 0.0247)	-6.95	0.0000
Parabacteroides	OW/OB	0.36	(0.0967, 1.3649)	-1.50	0.2100
Prevotella	GDM	7.13	(1.0323, 49.2806)	1.99	0.0792
Prevotella	OW/OB	70.17	(11.9506, 412.0464)	4.71	0.0000
Alistipes	GDM	0.03	(0.0056, 0.1405)	-4.35	0.0000
Alistipes	OW/OB	0.57	(0.1316, 2.4789)	-0.75	0.5735
Streptococcus	GDM	0.99	(0.4515, 2.1592)	-0.03	0.9746
Streptococcus	OW/OB	0.41	(0.2004, 0.8382)	-2.44	0.0281
Clostridium	GDM	0.71	(0.173, 2.9032)	-0.48	0.7234
Clostridium	OW/OB	0.04	(0.012, 0.158)	-4.76	0.0000
Finegoldia	GDM	0.14	(0.0187, 1.0413)	-1.92	0.0913

Finegoldia	OW/OB	22.59	(3.715, 137.3725)	3.38	0.0017
Peptoniphilus	GDM	2.40	(0.2229, 25.7465)	0.72	0.5840
Peptoniphilus	OW/OB	178.81	(20.2788, 1576.7166)	4.67	0.0000
Anaerostipes	GDM	14.29	(1.7131, 119.2339)	2.46	0.0274
Anaerostipes	OW/OB	28.39	(4.0603, 198.5558)	3.37	0.0017
Citrobacter	GDM	0.02	(0.0037, 0.0825)	-5.12	0.0000
Citrobacter	OW/OB	0.19	(0.0473, 0.7846)	-2.30	0.0404
Cronobacter	GDM	0.03	(0.0021, 0.4663)	-2.51	0.0238
Cronobacter	OW/OB	150.21	(16.741, 1347.828)	4.48	0.0000
Enterobacter	GDM	0.13	(0.035, 0.506)	-2.96	0.0067
Enterobacter	OW/OB	0.62	(0.1834, 2.106)	-0.76	0.5721
Raoultella	GDM	0.12	(0.0055, 2.6903)	-1.33	0.2735
Raoultella	OW/OB	0.02	(0.0014, 0.3351)	-2.74	0.0124
Serratia	GDM	15.26	(0.51, 456.362)	1.57	0.1843
Serratia	OW/OB	66.71	(2.6409, 1685.0298)	2.55	0.0217
Haemophilus	GDM	4.08	(1.0003, 16.6179)	1.96	0.0843
Haemophilus	OW/OB	0.22	(0.0596, 0.7791)	-2.34	0.0366
Acinetobacter	GDM	0.00	(0.0032, 0.0051)	-45.82	0.0000
Acinetobacter	OW/OB	3134.38	(2133.8136, 4604.1077)	41.03	0.0000
Unclassified *Christensenellaceae*	GDM	0.6383	(0.158, 2.5783)	-0.63	0.6287
Unclassified *Christensenellaceae*	OW/OB	7.0576	(1.9929, 24.9941)	3.03	0.0055
Unclassified *Lachnospiraceae*	GDM	5.5735	(1.772, 17.531)	2.94	0.0070
Unclassified *Lachnospiraceae*	OW/OB	12.6622	(4.44, 36.1102)	4.75	0.0000
Unclassified *Peptostreptococcaceae*	GDM	0.6279	(0.1768, 2.2292)	-0.72	0.5810
Unclassified *Peptostreptococcaceae*	OW/OB	105.1207	(33.1272, 333.5738)	7.90	0.0000
Unclassified *Ruminococcaceae*	GDM	0.7254	(0.2295, 2.2929)	-0.55	0.6851
Unclassified *Ruminococcaceae*	OW/OB	3.3653	(1.1755, 9.6344)	2.26	0.0437
Unclassified *Veillonellaceae*	GDM	0.5723	(0.0467, 7.0071)	-0.44	0.7432
Unclassified *Veillonellaceae*	OW/OB	27.8932	(2.8844, 269.7357)	2.88	0.0084

Rate ratios (RR) compared with NW/No GDM infant group from exponentiated beta coefficients. Wald confidence intervals for rate ratios. Z-score and FDR *p* value for original (non-exponentiated) beta coefficients.

Supplementary Table S12 Alpha and beta diversities by EWG and maternal overweight/obesity

	Group	Estimate difference in means	95% CI	*p* value
Alpha diversity				
Chao1	EWG	-0.3778	(-9.89,9.14)	0.937
Chao1	OW/OB	15.1789	(5.99,24.37)	0.002
Chao1	EWG:OW/OB	-10.3651	(-24.69,3.96)	0.152
Shannon $\frac{H}{H_{max}}$	EWG	0.0658	(-0.07,0.2)	0.322
Shannon $\frac{H}{H_{max}}$	OW/OB	0.1412	(0.01,0.27)	0.031

Shannon $\frac{H}{H_{max}}$	EWG:OW/OB	-0.0937	(-0.29,0.11)	0.348
Shannon H	EWG	0.2372	(-0.24,0.71)	0.320
Shannon H	OW/OB	0.5082	(0.05,0.97)	0.031
Shannon H	EWG:OW/OB	-0.3378	(-1.05,0.38)	0.347
Beta diversity				
Bray-Curtis	EWG	--	--	0.487
Bray-Curtis	OW/OB	--	--	0.043
Bray-Curtis	EWG:OW/OB	--	--	0.529
UniFrac				
Weighted	EWG	--	--	0.478
Weighted	OW/OB	--	--	0.227
Weighted	EWG:OW/OB	--	--	0.703

p values assessed by *t*-test with 42 degrees of freedom for alpha diversity.
p value assessed by PERMANOVA with 999 permutations for beta diversity and UniFrac.

Supplementary Table S13 Family-level taxa average relative abundances by maternal EWG status and BMI

Taxa	EWG	BMI group	RA
Actinomycetaceae	No EWG	NW	0.2931 (0.73)
Actinomycetaceae	No EWG	OW/OB	0.2215 (0.46)
Actinomycetaceae	EWG	NW	0.1377 (0.17)
Actinomycetaceae	EWG	OW/OB	0.0228 (0.05)
Alcaligenaceae	No EWG	NW	0.001 (0)
Alcaligenaceae	No EWG	OW/OB	0.091 (0.28)
Alcaligenaceae	EWG	NW	0.0586 (0.18)
Alcaligenaceae	EWG	OW/OB	0.2036 (0.61)
Bacillaceae	No EWG	NW	7e-04 (0)
Bacillaceae	No EWG	OW/OB	0.4568 (1.44)
Bacillaceae	EWG	NW	6e-04 (0)
Bacillaceae	EWG	OW/OB	0 (0)
Bacteroidaceae	No EWG	NW	28.8635 (31.25)
Bacteroidaceae	No EWG	OW/OB	13.5612 (24.23)
Bacteroidaceae	EWG	NW	16.8429 (26.42)
Bacteroidaceae	EWG	OW/OB	22.8008 (25.3)
Bifidobacteriaceae	No EWG	NW	4.3687 (9)
Bifidobacteriaceae	No EWG	OW/OB	14.017 (11.54)
Bifidobacteriaceae	EWG	NW	19.8357 (24.43)
Bifidobacteriaceae	EWG	OW/OB	19.5852 (25.96)
Christensenellaceae	No EWG	NW	0.0022 (0)
Christensenellaceae	No EWG	OW/OB	0.025 (0.07)
Christensenellaceae	EWG	NW	0.0011 (0)

Christensenellaceae	EWG	OW/OB	0.0025 (0)
Clostridiaceae	No EWG	NW	4.7241 (9.15)
Clostridiaceae	No EWG	OW/OB	0.0586 (0.06)
Clostridiaceae	EWG	NW	1.3443 (3.8)
Clostridiaceae	EWG	OW/OB	0.228 (0.36)
Coriobacteriaceae	No EWG	NW	0.2162 (0.67)
Coriobacteriaceae	No EWG	OW/OB	1.2922 (2.7)
Coriobacteriaceae	EWG	NW	0.9754 (1.35)
Coriobacteriaceae	EWG	OW/OB	0.4923 (1.35)
Corynebacteriaceae	No EWG	NW	0.0115 (0.01)
Corynebacteriaceae	No EWG	OW/OB	0.0423 (0.04)
Corynebacteriaceae	EWG	NW	0.2218 (0.58)
Corynebacteriaceae	EWG	OW/OB	0.0247 (0.02)
Desulfovibrionaceae	No EWG	NW	0.0739 (0.17)
Desulfovibrionaceae	No EWG	OW/OB	0.001 (0)
Desulfovibrionaceae	EWG	NW	5e-04 (0)
Desulfovibrionaceae	EWG	OW/OB	0.5988 (1.79)
Enterobacteriaceae	No EWG	NW	23.8608 (26.51)
Enterobacteriaceae	No EWG	OW/OB	32.1645 (24.58)
Enterobacteriaceae	EWG	NW	17.9891 (26.41)
Enterobacteriaceae	EWG	OW/OB	24.0086 (25.65)
Enterococcaceae	No EWG	NW	0.1049 (0.32)
Enterococcaceae	No EWG	OW/OB	0.2137 (0.58)
Enterococcaceae	EWG	NW	0.2628 (0.77)
Enterococcaceae	EWG	OW/OB	0.5619 (1.02)
Erysipelotrichaceae	No EWG	NW	0.7791 (3.27)
Erysipelotrichaceae	No EWG	OW/OB	0.5979 (1.05)
Erysipelotrichaceae	EWG	NW	1.873 (4.7)
Erysipelotrichaceae	EWG	OW/OB	0.385 (1.12)
Family-XIII-Incertae-Sedis	No EWG	NW	3e-04 (0)
Family-XIII-Incertae-Sedis	No EWG	OW/OB	0.0708 (0.22)
Family-XIII-Incertae-Sedis	EWG	NW	3e-04 (0)
Family-XIII-Incertae-Sedis	EWG	OW/OB	1e-04 (0)
Lachnospiraceae	No EWG	NW	1.2833 (3.16)
Lachnospiraceae	No EWG	OW/OB	11.2435 (21.46)
Lachnospiraceae	EWG	NW	0.3274 (0.79)
Lachnospiraceae	EWG	OW/OB	5.3536 (8.22)
Lactobacillaceae	No EWG	NW	0.3829 (1.16)
Lactobacillaceae	No EWG	OW/OB	0.8708 (1.73)
Lactobacillaceae	EWG	NW	3.0919 (5.38)
Lactobacillaceae	EWG	OW/OB	0.6224 (1.84)
Micrococcaceae	No EWG	NW	0.0656 (0.14)

Micrococcaceae	No EWG	OW/OB	0.0198 (0.03)
Micrococcaceae	EWG	NW	0.4652 (1.26)
Micrococcaceae	EWG	OW/OB	0.1619 (0.3)
Moraxellaceae	No EWG	NW	4e-04 (0)
Moraxellaceae	No EWG	OW/OB	0.0996 (0.29)
Moraxellaceae	EWG	NW	6e-04 (0)
Moraxellaceae	EWG	OW/OB	1.6043 (4.81)
Negativicoccus	No EWG	NW	0 (0)
Negativicoccus	No EWG	OW/OB	0.0915 (0.29)
Negativicoccus	EWG	NW	0 (0)
Negativicoccus	EWG	OW/OB	0.0021 (0.01)
Neisseriaceae	No EWG	NW	0.0019 (0)
Neisseriaceae	No EWG	OW/OB	0.016 (0.03)
Neisseriaceae	EWG	NW	0.0208 (0.04)
Neisseriaceae	EWG	OW/OB	0.0612 (0.14)
Pasteurellaceae	No EWG	NW	1.9002 (6.03)
Pasteurellaceae	No EWG	OW/OB	0.1943 (0.16)
Pasteurellaceae	EWG	NW	3.2437 (7.83)
Pasteurellaceae	EWG	OW/OB	1.2761 (2.93)
Peptostreptococcaceae	No EWG	NW	0.0069 (0.02)
Peptostreptococcaceae	No EWG	OW/OB	1.2562 (2.73)
Peptostreptococcaceae	EWG	NW	0.0075 (0.01)
Peptostreptococcaceae	EWG	OW/OB	0.0156 (0.03)
Porphyromonadaceae	No EWG	NW	0.4629 (1.91)
Porphyromonadaceae	No EWG	OW/OB	0.2634 (0.72)
Porphyromonadaceae	EWG	NW	6.7812 (13.94)
Porphyromonadaceae	EWG	OW/OB	0.7356 (1.44)
Prevotellaceae	No EWG	NW	0.0112 (0.04)
Prevotellaceae	No EWG	OW/OB	0.3258 (0.78)
Prevotellaceae	EWG	NW	6e-04 (0)
Prevotellaceae	EWG	OW/OB	0.0057 (0.01)
Propionibacteriaceae	No EWG	NW	0.2193 (0.84)
Propionibacteriaceae	No EWG	OW/OB	0.0647 (0.12)
Propionibacteriaceae	EWG	NW	1.5805 (4.74)
Propionibacteriaceae	EWG	OW/OB	0.0282 (0.07)
Rikenellaceae	No EWG	NW	0.3879 (1.61)
Rikenellaceae	No EWG	OW/OB	0.1849 (0.43)
Rikenellaceae	EWG	NW	0.0226 (0.06)
Rikenellaceae	EWG	OW/OB	0.0022 (0)
Ruminococcaceae	No EWG	NW	0.0991 (0.18)
Ruminococcaceae	No EWG	OW/OB	0.2927 (0.51)
Ruminococcaceae	EWG	NW	0.0279 (0.06)

Ruminococcaceae	EWG	OW/OB	0.2224 (0.56)
Staphylococcaceae	No EWG	NW	4.249 (7.63)
Staphylococcaceae	No EWG	OW/OB	2.5545 (5.28)
Staphylococcaceae	EWG	NW	4.1494 (8.64)
Staphylococcaceae	EWG	OW/OB	3.3516 (3.57)
Streptococcaceae	No EWG	NW	15.8575 (22.51)
Streptococcaceae	No EWG	OW/OB	5.869 (5.76)
Streptococcaceae	EWG	NW	9.0019 (12.97)
Streptococcaceae	EWG	OW/OB	5.1915 (5.05)
Unclassified *Bacillales*	No EWG	NW	0.0922 (0.34)
Unclassified *Bacillales*	No EWG	OW/OB	0.0156 (0.03)
Unclassified *Bacillales*	EWG	NW	0.0191 (0.05)
Unclassified *Bacillales*	EWG	OW/OB	0.0103 (0.01)
Unclassified *Bacteroidales*	No EWG	NW	0.4497 (1.4)
Unclassified *Bacteroidales*	No EWG	OW/OB	0.0705 (0.12)
Unclassified *Bacteroidales*	EWG	NW	0.6098 (1.51)
Unclassified *Bacteroidales*	EWG	OW/OB	3.5492 (5.06)
Unclassified *Clostridiales*	No EWG	NW	3.0438 (7.47)
Unclassified *Clostridiales*	No EWG	OW/OB	1.045 (1.99)
Unclassified *Clostridiales*	EWG	NW	1.5647 (2.93)
Unclassified *Clostridiales*	EWG	OW/OB	1.8055 (3.51)
Unclassified *Lactobacillales*	No EWG	NW	0.1106 (0.33)
Unclassified *Lactobacillales*	No EWG	OW/OB	0.0175 (0.03)
Unclassified *Lactobacillales*	EWG	NW	8e-04 (0)
Unclassified *Lactobacillales*	EWG	OW/OB	0.0071 (0.01)
Unclassified *Selenomonadales*	No EWG	NW	0.0083 (0.04)
Unclassified *Selenomonadales*	No EWG	OW/OB	1e-04 (0)
Unclassified *Selenomonadales*	EWG	NW	0 (0)
Unclassified *Selenomonadales*	EWG	OW/OB	1e-04 (0)
Veillonellaceae	No EWG	NW	8e-04 (0)
Veillonellaceae	No EWG	OW/OB	0.0386 (0.12)
Veillonellaceae	EWG	NW	2e-04 (0)
Veillonellaceae	EWG	OW/OB	1e-04 (0)
Xanthomonadaceae	No EWG	NW	0 (0)
Xanthomonadaceae	No EWG	OW/OB	0.0545 (0.17)
Xanthomonadaceae	EWG	NW	0.0481 (0.14)
Xanthomonadaceae	EWG	OW/OB	0 (0)
Other	No EWG	NW	0.0195 (0.03)
Other	No EWG	OW/OB	0.1327 (0.28)
Other	EWG	NW	0.0207 (0.03)
Other	EWG	OW/OB	0.0222 (0.02)

Data expressed as mean (SD).

Supplementary Table S14 Genus-level taxa average relative abundances by maternal EWG status and BMI

Taxa	EWG	BMI group	RA
Acinetobacter	No EWG	NW	3e-04 (0)
Acinetobacter	No EWG	OW/OB	0.0993 (0.29)
Acinetobacter	EWG	NW	6e-04 (0)
Acinetobacter	EWG	OW/OB	1.6043 (4.81)
Actinomyces	No EWG	NW	0.2909 (0.73)
Actinomyces	No EWG	OW/OB	0.221 (0.46)
Actinomyces	EWG	NW	0.1377 (0.17)
Actinomyces	EWG	OW/OB	0.0199 (0.05)
Alistipes	No EWG	NW	0.3876 (1.61)
Alistipes	No EWG	OW/OB	0.184 (0.43)
Alistipes	EWG	NW	0.0224 (0.06)
Alistipes	EWG	OW/OB	0.0021 (0)
Anaerostipes	No EWG	NW	0.0037 (0.01)
Anaerostipes	No EWG	OW/OB	0.5878 (1.76)
Anaerostipes	EWG	NW	0 (0)
Anaerostipes	EWG	OW/OB	0.0011 (0)
Anaerotruncus	No EWG	NW	1e-04 (0)
Anaerotruncus	No EWG	OW/OB	0.153 (0.48)
Anaerotruncus	EWG	NW	2e-04 (0)
Anaerotruncus	EWG	OW/OB	5e-04 (0)
Atopobium	No EWG	NW	0.001 (0)
Atopobium	No EWG	OW/OB	0.0566 (0.15)
Atopobium	EWG	NW	0.2921 (0.83)
Atopobium	EWG	OW/OB	0.0088 (0.02)
Bacteroides	No EWG	NW	28.8635 (31.25)
Bacteroides	No EWG	OW/OB	13.5612 (24.23)
Bacteroides	EWG	NW	16.8429 (26.42)
Bacteroides	EWG	OW/OB	22.8008 (25.3)
Bifidobacterium	No EWG	NW	4.3686 (9)
Bifidobacterium	No EWG	OW/OB	14.01 (11.53)
Bifidobacterium	EWG	NW	19.8356 (24.43)
Bifidobacterium	EWG	OW/OB	19.5852 (25.96)
Bilophila	No EWG	NW	0.0737 (0.17)
Bilophila	No EWG	OW/OB	0.001 (0)
Bilophila	EWG	NW	4e-04 (0)
Bilophila	EWG	OW/OB	0.3853 (1.15)
Blautia	No EWG	NW	0.0066 (0.01)
Blautia	No EWG	OW/OB	0.8893 (2.79)
Blautia	EWG	NW	0.0082 (0.02)

Blautia	EWG	OW/OB	0.0048 (0.01)
Butyricimonas	No EWG	NW	0 (0)
Butyricimonas	No EWG	OW/OB	3e-04 (0)
Butyricimonas	EWG	NW	0.0579 (0.17)
Butyricimonas	EWG	OW/OB	0 (0)
Citrobacter	No EWG	NW	0.0066 (0.01)
Citrobacter	No EWG	OW/OB	0.0719 (0.22)
Citrobacter	EWG	NW	1.4072 (4.2)
Citrobacter	EWG	OW/OB	0.0045 (0.01)
Clostridium	No EWG	NW	4.4914 (9.15)
Clostridium	No EWG	OW/OB	0.0448 (0.05)
Clostridium	EWG	NW	1.31 (3.73)
Clostridium	EWG	OW/OB	0.2267 (0.36)
Collinsella	No EWG	NW	0.1196 (0.49)
Collinsella	No EWG	OW/OB	1.0087 (2.78)
Collinsella	EWG	NW	0.3167 (0.95)
Collinsella	EWG	OW/OB	0.0015 (0)
Corynebacterium	No EWG	NW	0.0102 (0.01)
Corynebacterium	No EWG	OW/OB	0.0228 (0.02)
Corynebacterium	EWG	NW	0.2171 (0.58)
Corynebacterium	EWG	OW/OB	0.0188 (0.02)
Cronobacter	No EWG	NW	1e-04 (0)
Cronobacter	No EWG	OW/OB	0.1481 (0.47)
Cronobacter	EWG	NW	7e-04 (0)
Cronobacter	EWG	OW/OB	0.0024 (0.01)
Desulfovibrio	No EWG	NW	2e-04 (0)
Desulfovibrio	No EWG	OW/OB	0 (0)
Desulfovibrio	EWG	NW	1e-04 (0)
Desulfovibrio	EWG	OW/OB	0.2134 (0.64)
Dorea	No EWG	NW	7e-04 (0)
Dorea	No EWG	OW/OB	0.3701 (1.17)
Dorea	EWG	NW	1e-04 (0)
Dorea	EWG	OW/OB	0 (0)
Eggerthella	No EWG	NW	0.0253 (0.1)
Eggerthella	No EWG	OW/OB	0.0642 (0.1)
Eggerthella	EWG	NW	6e-04 (0)
Eggerthella	EWG	OW/OB	0.1189 (0.31)
Enterobacter	No EWG	NW	0.7997 (2.62)
Enterobacter	No EWG	OW/OB	0.1057 (0.22)
Enterobacter	EWG	NW	1.2238 (3.5)
Enterobacter	EWG	OW/OB	0.4576 (0.93)
Enterococcus	No EWG	NW	0.1049 (0.32)

Enterococcus	No EWG	OW/OB	0.2137 (0.58)
Enterococcus	EWG	NW	0.2628 (0.77)
Enterococcus	EWG	OW/OB	0.5619 (1.02)
Escherichia-Shigella	No EWG	NW	11.8141 (15.89)
Escherichia-Shigella	No EWG	OW/OB	20.8983 (21.82)
Escherichia-Shigella	EWG	NW	10.6822 (26.5)
Escherichia-Shigella	EWG	OW/OB	14.7972 (20.39)
Faecalibacterium	No EWG	NW	0.0075 (0.02)
Faecalibacterium	No EWG	OW/OB	0.0179 (0.04)
Faecalibacterium	EWG	NW	0.0017 (0)
Faecalibacterium	EWG	OW/OB	0.0043 (0.01)
Finegoldia	No EWG	NW	0.002 (0.01)
Finegoldia	No EWG	OW/OB	0.0101 (0.02)
Finegoldia	EWG	NW	0.0044 (0.01)
Finegoldia	EWG	OW/OB	0.0892 (0.23)
Flavonifractor	No EWG	NW	0.0172 (0.07)
Flavonifractor	No EWG	OW/OB	0.0023 (0)
Flavonifractor	EWG	NW	0.0211 (0.06)
Flavonifractor	EWG	OW/OB	6e-04 (0)
Gemella	No EWG	NW	0.0287 (0.04)
Gemella	No EWG	OW/OB	0.0881 (0.12)
Gemella	EWG	NW	0.0476 (0.06)
Gemella	EWG	OW/OB	0.0488 (0.11)
Haemophilus	No EWG	NW	1.9001 (6.03)
Haemophilus	No EWG	OW/OB	0.1942 (0.16)
Haemophilus	EWG	NW	3.2417 (7.83)
Haemophilus	EWG	OW/OB	1.2745 (2.93)
Klebsiella	No EWG	NW	0.0019 (0.01)
Klebsiella	No EWG	OW/OB	0.0127 (0.04)
Klebsiella	EWG	NW	0.0051 (0.02)
Klebsiella	EWG	OW/OB	6e-04 (0)
Lactobacillus	No EWG	NW	0.3829 (1.16)
Lactobacillus	No EWG	OW/OB	0.8708 (1.73)
Lactobacillus	EWG	NW	3.0919 (5.38)
Lactobacillus	EWG	OW/OB	0.6224 (1.84)
Negativicoccus	No EWG	NW	0 (0)
Negativicoccus	No EWG	OW/OB	0.0915 (0.29)
Negativicoccus	EWG	NW	0 (0)
Negativicoccus	EWG	OW/OB	0.0021 (0.01)
Neisseria	No EWG	NW	0.0016 (0)
Neisseria	No EWG	OW/OB	0.0125 (0.03)
Neisseria	EWG	NW	0.0208 (0.04)

Neisseria	EWG	OW/OB	0.0612 (0.14)
Pantoea	No EWG	NW	0.7737 (3.05)
Pantoea	No EWG	OW/OB	0 (0)
Pantoea	EWG	NW	1e-04 (0)
Pantoea	EWG	OW/OB	0 (0)
Parabacteroides	No EWG	NW	0.462 (1.91)
Parabacteroides	No EWG	OW/OB	0.0932 (0.22)
Parabacteroides	EWG	NW	6.7232 (13.89)
Parabacteroides	EWG	OW/OB	0.7331 (1.44)
Peptoniphilus	No EWG	NW	3e-04 (0)
Peptoniphilus	No EWG	OW/OB	0.0121 (0.02)
Peptoniphilus	EWG	NW	3e-04 (0)
Peptoniphilus	EWG	OW/OB	0.0685 (0.18)
Phascolarctobacterium	No EWG	NW	0.1283 (0.54)
Phascolarctobacterium	No EWG	OW/OB	0.0104 (0.03)
Phascolarctobacterium	EWG	NW	9e-04 (0)
Phascolarctobacterium	EWG	OW/OB	0.0016 (0)
Porphyromonas	No EWG	NW	0 (0)
Porphyromonas	No EWG	OW/OB	0.1692 (0.5)
Porphyromonas	EWG	NW	0 (0)
Porphyromonas	EWG	OW/OB	3e-04 (0)
Prevotella	No EWG	NW	0.0111 (0.04)
Prevotella	No EWG	OW/OB	0.3251 (0.78)
Prevotella	EWG	NW	3e-04 (0)
Prevotella	EWG	OW/OB	0.0055 (0.01)
Propionibacterium	No EWG	NW	0.2193 (0.84)
Propionibacterium	No EWG	OW/OB	0.0643 (0.12)
Propionibacterium	EWG	NW	1.5805 (4.74)
Propionibacterium	EWG	OW/OB	0.0279 (0.07)
Raoultella	No EWG	NW	0.026 (0.11)
Raoultella	No EWG	OW/OB	1e-04 (0)
Raoultella	EWG	NW	0 (0)
Raoultella	EWG	OW/OB	2e-04 (0)
Rothia	No EWG	NW	0.0655 (0.14)
Rothia	No EWG	OW/OB	0.0187 (0.03)
Rothia	EWG	NW	0.465 (1.26)
Rothia	EWG	OW/OB	0.1619 (0.3)
Serratia	No EWG	NW	0 (0)
Serratia	No EWG	OW/OB	0 (0)
Serratia	EWG	NW	1e-04 (0)
Serratia	EWG	OW/OB	0.0414 (0.12)
Staphylococcus	No EWG	NW	4.249 (7.63)

Staphylococcus	No EWG	OW/OB	2.5545 (5.28)
Staphylococcus	EWG	NW	4.1494 (8.64)
Staphylococcus	EWG	OW/OB	3.3516 (3.57)
Stenotrophomonas	No EWG	NW	0 (0)
Stenotrophomonas	No EWG	OW/OB	0.0545 (0.17)
Stenotrophomonas	EWG	NW	0.0481 (0.14)
Stenotrophomonas	EWG	OW/OB	0 (0)
Streptococcus	No EWG	NW	15.8428 (22.49)
Streptococcus	No EWG	OW/OB	5.8588 (5.75)
Streptococcus	EWG	NW	8.907 (12.96)
Streptococcus	EWG	OW/OB	5.1787 (5.05)
Sutterella	No EWG	NW	5e-04 (0)
Sutterella	No EWG	OW/OB	0.0902 (0.29)
Sutterella	EWG	NW	0.0584 (0.17)
Sutterella	EWG	OW/OB	0.2035 (0.61)
Unclassified *Christensenellaceae*	No EWG	NW	0.0022 (0)
Unclassified *Christensenellaceae*	No EWG	OW/OB	0.025 (0.07)
Unclassified *Christensenellaceae*	EWG	NW	0.0011 (0)
Unclassified *Christensenellaceae*	EWG	OW/OB	0.0025 (0)
Unclassified *Clostridiaceae*	No EWG	NW	0.2327 (0.68)
Unclassified *Clostridiaceae*	No EWG	OW/OB	0.0138 (0.04)
Unclassified *Clostridiaceae*	EWG	NW	0.0342 (0.07)
Unclassified *Clostridiaceae*	EWG	OW/OB	0.0013 (0)
Unclassified *Coriobacteriaceae*	No EWG	NW	0.0676 (0.18)
Unclassified *Coriobacteriaceae*	No EWG	OW/OB	0.1587 (0.32)
Unclassified *Coriobacteriaceae*	EWG	NW	0.2936 (0.61)
Unclassified *Coriobacteriaceae*	EWG	OW/OB	0.3572 (1.01)
Unclassified *Enterobacteriaceae*	No EWG	NW	10.4384 (19.63)
Unclassified *Enterobacteriaceae*	No EWG	OW/OB	10.9271 (13.72)
Unclassified *Enterobacteriaceae*	EWG	NW	4.67 (7.21)
Unclassified *Enterobacteriaceae*	EWG	OW/OB	8.7025 (9.48)
Unclassified *Erysipelotrichaceae*	No EWG	NW	0.7789 (3.27)
Unclassified *Erysipelotrichaceae*	No EWG	OW/OB	0.5962 (1.05)
Unclassified *Erysipelotrichaceae*	EWG	NW	1.8722 (4.7)
Unclassified *Erysipelotrichaceae*	EWG	OW/OB	0.3834 (1.12)
Unclassified *Family-XIII-Incertae-Sedis*	No EWG	NW	3e-04 (0)
Unclassified *Family-XIII-Incertae-Sedis*	No EWG	OW/OB	0.0708 (0.22)
Unclassified *Family-XIII-Incertae-Sedis*	EWG	NW	3e-04 (0)
Unclassified *Family-XIII-Incertae-Sedis*	EWG	OW/OB	1e-04 (0)
Unclassified *Lachnospiraceae*	No EWG	NW	1.2632 (3.16)
Unclassified *Lachnospiraceae*	No EWG	OW/OB	9.3812 (16.35)
Unclassified *Lachnospiraceae*	EWG	NW	0.3162 (0.8)

Unclassified *Lachnospiraceae*	EWG	OW/OB	5.3415 (8.22)
Unclassified *Peptostreptococcaceae*	No EWG	NW	0.0069 (0.02)
Unclassified *Peptostreptococcaceae*	No EWG	OW/OB	1.2548 (2.73)
Unclassified *Peptostreptococcaceae*	EWG	NW	0.0075 (0.01)
Unclassified *Peptostreptococcaceae*	EWG	OW/OB	0.0156 (0.03)
Unclassified *Ruminococcaceae*	No EWG	NW	0.0673 (0.15)
Unclassified *Ruminococcaceae*	No EWG	OW/OB	0.1135 (0.22)
Unclassified *Ruminococcaceae*	EWG	NW	0.0041 (0)
Unclassified *Ruminococcaceae*	EWG	OW/OB	0.2116 (0.56)
Unclassified *Streptococcaceae*	No EWG	NW	0.0147 (0.03)
Unclassified *Streptococcaceae*	No EWG	OW/OB	0.0099 (0.01)
Unclassified *Streptococcaceae*	EWG	NW	0.0934 (0.24)
Unclassified *Streptococcaceae*	EWG	OW/OB	0.0127 (0.02)
Unclassified *Veillonellaceae*	No EWG	NW	8e-04 (0)
Unclassified *Veillonellaceae*	No EWG	OW/OB	0.0386 (0.12)
Unclassified *Veillonellaceae*	EWG	NW	2e-04 (0)
Unclassified *Veillonellaceae*	EWG	OW/OB	1e-04 (0)
Veillonella	No EWG	NW	6.3014 (11.66)
Veillonella	No EWG	OW/OB	8.4947 (12.75)
Veillonella	EWG	NW	7.2729 (9.89)
Veillonella	EWG	OW/OB	5.5769 (14)
Other	No EWG	NW	0.0452 (0.06)
Other	No EWG	OW/OB	0.6541 (1.76)
Other	EWG	NW	0.1077 (0.22)
Other	EWG	OW/OB	0.0571 (0.05)

Data expressed as mean (SD).

Supplementary Table S15 Summary of maternal EWG impact on infant microbial family- and genus-level taxa

**EWG + NW***		**EWG#**	
Increase	Decrease	Increase	Decrease
**Family**			
Corynebacteriaceae	Unclassified *Lactobacillales*	*Micrococcaceae*	*Prevotellaceae*
Moraxellaceae	*Family-XIII-Incertae-Sedis*	*Porphyromonadaceae*	*Rikenellaceae*
Neisseriaceae		*Alcaligenaceae*	*Veillonellaceae*
Peptostreptococcaceae			
Xanthomonadaceae			
Unclassified *Bacteroidales*			
**Genus**			
Acinetobacter	*Blautia*	*Rothia*	*Prevotella*
Anaerotruncus	*Klebsiella*	*Parabacteroides*	*Alistipes*
Corynebacterium	*Raoultella*	Unclassified *Streptococcaceae*	*Anaerostipes*
Klebsiella	Unclassified *Family-XIII-Incertae-Sedis*		*Phascolarctobacterium*

Neisseria	Unclassified *Clostridiaceae*
Stenotrophomonas	Unclassified *Veillonellaceae*
Unclassified *Peptostreptococcaceae*	
Unclassified *Ruminococcaceae*	

*Determined by FDR *p* value < 0.05 in model with an interaction between maternal EWG and OW/OB (i.e., relative abundance differences between EWG and no EWG in offspring from NW mothers only).
#Determined by FDR *p* value < 0.05 in an additive model controlling for maternal OW/OB (i.e., relative abundance differences in EWG that have the same impact on offspring from OW/OB and NW mothers).

Supplementary Table S16 Phylum-level taxa negative binomial models with significant EWG:OW/OB interactions

Taxa	Group	RR	95% CI	Z	FDR *p* value
Fusobacteria	EWG	10.36	(1.1178, 96.0823)	2.06	0.0864
Fusobacteria	OW/OB	137.38	(16.0794, 1173.7993)	4.50	< 0.0001
Fusobacteria	EWG:OW/OB	0.54	(0.0457, 6.4569)	-4.44	< 0.0001

Rate ratios (RR) compared with NW/No EWG infant group from exponentiated beta coefficients or sums of beta coefficients for interaction term (EWG:OW/OB). Wald confidence intervals for rate ratios. Z-score and FDR *p* value for original (non-exponentiated) beta coefficients.

Supplementary Table S17 Family-level taxa negative binomial models with significant EWG:OW/OB interactions

Taxa	Group	RR	95% CI	Z	FDR *p* value
Corynebacteriaceae	EWG	19.33	(6.7479, 55.3651)	5.52	< 0.0001
Corynebacteriaceae	OW/OB	3.68	(1.3262, 10.1858)	2.50	0.0253
Corynebacteriaceae	EWG:OW/OB	2.14	(0.7441, 6.1787)	-4.33	< 0.0001
Family-XIII-Incertae-Sedis	EWG	0.90	(0.0784, 10.2767)	-0.09	0.95
Family-XIII-Incertae-Sedis	OW/OB	253.39	(29.1983, 2199.0085)	5.02	< 0.0001
Family-XIII-Incertae-Sedis	EWG:OW/OB	0.47	(0.0317, 7.11)	-3.22	0.0033
Peptostreptococcaceae	EWG	1.08	(0.272, 4.273)	0.11	0.9455
Peptostreptococcaceae	OW/OB	181.88	(48.6243, 680.3162)	7.73	< 0.0001
Peptostreptococcaceae	EWG:OW/OB	2.23	(0.5651, 8.804)	-4.25	0.0001
Neisseriaceae	EWG	12.12	(9.4439, 15.5673)	19.57	< 0.0001
Neisseriaceae	OW/OB	9.61	(7.4543, 12.4017)	17.43	< 0.0001
Neisseriaceae	EWG:OW/OB	31.31	(24.6368, 39.7824)	-9.15	< 0.0001
Moraxellaceae	EWG	0.79	(0.335, 1.8738)	-0.53	0.6639
Moraxellaceae	OW/OB	252.10	(162.3318, 391.5191)	24.62	< 0.0001
Moraxellaceae	EWG:OW/OB	5085.41	(3280.46, 7883.4638)	7.36	< 0.0001
Xanthomonadaceae	EWG	1025.53	(144.142, 7296.3085)	6.93	< 0.0001
Xanthomonadaceae	OW/OB	2836.30	(399.2101, 20151.28)	7.95	< 0.0001
Xanthomonadaceae	EWG:OW/OB	2.84	(0.1778, 45.4461)	-9.78	< 0.0001
Unclassified *Bacteroidales*	EWG	1.36	(0.3122, 5.8947)	0.41	0.7428
Unclassified *Bacteroidales*	OW/OB	0.16	(0.0379, 0.6489)	-2.56	0.0223
Unclassified *Bacteroidales*	EWG:OW/OB	7.90	(1.8171, 34.305)	3.20	0.0035
Unclassified *Lactobacillales*	EWG	0.01	(0.0011, 0.047)	-5.17	0.0000

Unclassified *Lactobacillales*	OW/OB	0.16	(0.0281, 0.8942)	-2.09	0.0693
Unclassified *Lactobacillales*	EWG:OW/OB	0.06	(0.0106, 0.3875)	2.86	0.0097

Rate ratios (RR) compared with NW/No EWG infant group from exponentiated beta coefficients or sums of beta coefficients for interaction term (EWG:OW/OB). Wald confidence intervals for rate ratios. Z-score and FDR *p* value for original (non-exponentiated) beta coefficients.

Supplementary Table S18 Genus-level taxa negative binomial models with significant EWG:OW/OB interactions

Taxa	Group	RR	95% CI	Z	FDR *p* value
Corynebacterium	EWG	21.41	(7.411, 61.8392)	5.66	0.0000
Corynebacterium	OW/OB	2.25	(0.8034, 6.3024)	1.54	0.1930
Corynebacterium	EWG:OW/OB	1.85	(0.6367, 5.3971)	-3.99	0.0002
Blautia	EWG	1.24	(0.2469, 6.2676)	0.26	0.8430
Blautia	OW/OB	136.12	(28.7903, 643.5281)	6.20	0.0000
Blautia	EWG:OW/OB	0.72	(0.1407, 3.6619)	-4.40	0.0000
Anaerotruncus	EWG	1.33	(0.1149, 15.2887)	0.23	0.8686
Anaerotruncus	OW/OB	1098.09	(134.3435, 8975.5318)	6.53	0.0000
Anaerotruncus	EWG:OW/OB	3.71	(0.3711, 37.0802)	-3.45	0.0014
Neisseria	EWG	14.28	(10.9207, 18.6627)	19.45	0.0000
Neisseria	OW/OB	9.96	(7.5627, 13.1155)	16.37	0.0000
Neisseria	EWG:OW/OB	36.86	(28.469, 47.7271)	-8.80	0.0000
Bilophila	EWG	0.01	(4e-04, 0.1012)	-3.56	0.0010
Bilophila	OW/OB	0.01	(9e-04, 0.1925)	-3.17	0.0034
Bilophila	EWG:OW/OB	5.23	(0.3496, 78.2001)	5.20	0.0000
Klebsiella	EWG	2.45	(1.8273, 3.274)	6.01	0.0000
Klebsiella	OW/OB	4.11	(3.167, 5.3356)	10.62	0.0000
Klebsiella	EWG:OW/OB	0.26	(0.1267, 0.5392)	-9.32	0.0000
Raoultella	EWG	0.00	(0, 0.0822)	-3.28	0.0026
Raoultella	OW/OB	0.00	(2e-04, 0.1238)	-3.23	0.0030
Raoultella	EWG:OW/OB	0.01	(3e-04, 0.2232)	2.47	0.0259
Acinetobacter	EWG	0.83	(0.3506, 1.984)	-0.41	0.7538
Acinetobacter	OW/OB	264.79	(168.5764, 415.9076)	24.22	0.0000
Acinetobacter	EWG:OW/OB	5352.97	(3413.9743, 8393.2321)	7.20	0.0000
Stenotrophomonas	EWG	1023.26	(143.8226, 7280.2116)	6.92	0.0000
Stenotrophomonas	OW/OB	2836.31	(399.2121, 20151.3809)	7.95	0.0000
Stenotrophomonas	EWG:OW/OB	2.84	(0.1778, 45.4461)	-9.78	0.0000
Unclassified *Family-XIII-Incertae-Sedis*	EWG	0.90	(0.0784, 10.2767)	-0.09	0.9510
Unclassified *Family-XIII-Incertae-Sedis*	OW/OB	253.39	(29.1983, 2199.0085)	5.02	0.0000
Unclassified *Family-XIII-Incertae-Sedis*	EWG:OW/OB	0.47	(0.0317, 7.11)	-3.22	0.0030
Unclassified *Peptostreptococcaceae*	EWG	1.08	(0.2687, 4.3244)	0.11	0.9406
Unclassified *Peptostreptococcaceae*	OW/OB	181.67	(48.0059, 687.5227)	7.66	0.0000
Unclassified *Peptostreptococcaceae*	EWG:OW/OB	2.23	(0.5585, 8.9123)	-4.21	0.0001
Unclassified *Ruminococcaceae*	EWG	0.06	(0.0159, 0.2306)	-4.11	0.0001
Unclassified *Ruminococcaceae*	OW/OB	1.69	(0.4737, 6.0043)	0.81	0.5158

Unclassified *Ruminococcaceae*	EWG:OW/OB	3.15	(0.8454, 11.7063)	3.37	0.0019

Rate ratios (RR) compared with NW/No EWG infant group from exponentiated beta coefficients or sums of beta coefficients for interaction term (EWG:OW/OB). Wald confidence intervals for rate ratios. Z-score and FDR *p* value for original (non-exponentiated) beta coefficients.

Supplementary Table S19 Family-level taxa negative binomial models with significant EWG or OW/OB main effects, controlling for other factor

Taxa	Group	RR	95% CI	Z	FDR *p* value
Micrococcaceae	EWG	7.55	(1.9322, 29.4928)	2.91	0.0080
Micrococcaceae	OW/OB	0.32	(0.083, 1.2382)	-1.65	0.1507
Propionibacteriaceae	EWG	2.65	(0.5121, 13.7505)	1.16	0.3222
Propionibacteriaceae	OW/OB	0.11	(0.0222, 0.579)	-2.62	0.0181
Porphyromonadaceae	EWG	7.58	(1.8631, 30.8496)	2.83	0.0100
Porphyromonadaceae	OW/OB	0.30	(0.0757, 1.2223)	-1.68	0.1448
Prevotellaceae	EWG	0.03	(0.0066, 0.1487)	-4.36	0.0000
Prevotellaceae	OW/OB	19.56	(4.2866, 89.2429)	3.84	0.0003
Rikenellaceae	EWG	0.03	(0.0077, 0.1236)	-4.92	0.0000
Rikenellaceae	OW/OB	0.26	(0.0664, 1.0342)	-1.91	0.0958
Streptococcaceae	EWG	0.69	(0.3396, 1.4152)	-1.01	0.3918
Streptococcaceae	OW/OB	0.45	(0.2195, 0.9036)	-2.24	0.0462
Christensenellaceae	EWG	0.25	(0.0722, 0.867)	-2.19	0.0523
Christensenellaceae	OW/OB	6.40	(1.9031, 21.5548)	3.00	0.0062
Clostridiaceae	EWG	1.02	(0.2863, 3.6456)	0.03	0.9737
Clostridiaceae	OW/OB	0.04	(0.0108, 0.1342)	-5.08	0.0000
Lachnospiraceae	EWG	0.34	(0.115, 0.9976)	-1.96	0.0864
Lachnospiraceae	OW/OB	11.38	(3.9012, 33.199)	4.45	0.0000
Ruminococcaceae	EWG	0.45	(0.1646, 1.2066)	-1.59	0.1625
Ruminococcaceae	OW/OB	4.49	(1.6743, 12.0534)	2.98	0.0064
Veillonellaceae	EWG	0.04	(0.0043, 0.3898)	-2.78	0.0113
Veillonellaceae	OW/OB	14.96	(1.7978, 124.5028)	2.50	0.0242
Alcaligenaceae	EWG	20.03	(2.8585, 140.3302)	3.02	0.0060
Alcaligenaceae	OW/OB	32.56	(4.7248, 224.4262)	3.54	0.0010
Pasteurellaceae	EWG	3.20	(0.8704, 11.794)	1.75	0.1258
Pasteurellaceae	OW/OB	0.18	(0.0498, 0.66)	-2.59	0.0192
Unclassified *Bacillales*	EWG	0.36	(0.1178, 1.0733)	-1.83	0.1093
Unclassified *Bacillales*	OW/OB	0.28	(0.0923, 0.8251)	-2.30	0.0401

Rate ratios (RR) compared with NW/No EWG infant group from exponentiated beta coefficients. Wald confidence intervals for rate ratios. Z-score and FDR *p* value for original (non-exponentiated) beta coefficients.

Supplementary Table S20 Genus-level taxa negative binomial models with significant EWG or OW/OB main effects, controlling for other factor

Taxa	Group	RR	95% CI	Z	FDR *p* value
Rothia	EWG	7.75	(1.9758, 30.4)	2.94	0.0074
Rothia	OW/OB	0.31	(0.08, 1.2029)	-1.69	0.1357

Propionibacterium	EWG	2.65	(0.5109, 13.7708)	1.16	0.3240
Propionibacterium	OW/OB	0.11	(0.022, 0.5757)	-2.62	0.0177
Eggerthella	EWG	0.24	(0.0339, 1.7378)	-1.41	0.2239
Eggerthella	OW/OB	14.95	(2.1295, 104.9657)	2.72	0.0137
Butyricimonas	EWG	1.00	(1, 1)	0.00	0.9993
Butyricimonas	OW/OB	0.00	(0, 0)	-86.08	0.0000
Parabacteroides	EWG	11.19	(2.7857, 44.9775)	3.40	0.0016
Parabacteroides	OW/OB	0.16	(0.0397, 0.6253)	-2.63	0.0177
Prevotella	EWG	0.02	(0.0039, 0.1138)	-4.48	0.0000
Prevotella	OW/OB	24.70	(4.816, 126.6859)	3.84	0.0003
Alistipes	EWG	0.03	(0.0072, 0.1285)	-4.75	0.0000
Alistipes	OW/OB	0.26	(0.0619, 1.0787)	-1.86	0.1029
Streptococcus	EWG	0.69	(0.3374, 1.4083)	-1.02	0.3884
Streptococcus	OW/OB	0.45	(0.22, 0.9069)	-2.23	0.0460
Clostridium	EWG	1.19	(0.3248, 4.3604)	0.26	0.8494
Clostridium	OW/OB	0.03	(0.0093, 0.1226)	-5.16	0.0000
Finegoldia	EWG	4.22	(0.6879, 25.9202)	1.56	0.1731
Finegoldia	OW/OB	9.01	(1.492, 54.4652)	2.40	0.0311
Peptoniphilus	EWG	2.18	(0.2488, 19.041)	0.70	0.5672
Peptoniphilus	OW/OB	83.04	(9.7416, 707.8457)	4.04	0.0001
Anaerostipes	EWG	0.00	(1e-04, 0.016)	-5.37	0.0000
Anaerostipes	OW/OB	177.21	(23.2853, 1348.682)	5.00	0.0000
Flavonifractor	EWG	0.68	(0.1114, 4.1959)	-0.41	0.7500
Flavonifractor	OW/OB	0.08	(0.0125, 0.4589)	-2.81	0.0109
Phascolarctobacterium	EWG	0.03	(0.0047, 0.2078)	-3.58	0.0008
Phascolarctobacterium	OW/OB	0.29	(0.0439, 1.8696)	-1.31	0.2641
Enterobacter	EWG	2.47	(0.7076, 8.6442)	1.42	0.2230
Enterobacter	OW/OB	0.21	(0.0594, 0.7098)	-2.50	0.0239
Haemophilus	EWG	3.20	(0.8658, 11.8427)	1.74	0.1233
Haemophilus	OW/OB	0.18	(0.0496, 0.6627)	-2.58	0.0196
Unclassified *Christensenellaceae*	EWG	0.25	(0.0722, 0.867)	-2.19	0.0515
Unclassified *Christensenellaceae*	OW/OB	6.40	(1.9031, 21.5548)	3.00	0.0062
Unclassified *Clostridiaceae*	EWG	0.12	(0.0191, 0.7472)	-2.27	0.0424
Unclassified *Clostridiaceae*	OW/OB	0.05	(0.0079, 0.2996)	-3.26	0.0026
Unclassified *Lachnospiraceae*	EWG	0.36	(0.1214, 1.0956)	-1.80	0.1125
Unclassified *Lachnospiraceae*	OW/OB	10.50	(3.529, 31.2276)	4.23	0.0001
Unclassified *Streptococcaceae*	EWG	3.38	(1.283, 8.9294)	2.46	0.0264
Unclassified *Streptococcaceae*	OW/OB	0.37	(0.1395, 0.9592)	-2.04	0.0718
Unclassified *Veillonellaceae*	EWG	0.04	(0.0043, 0.3898)	-2.78	0.0117
Unclassified *Veillonellaceae*	OW/OB	14.96	(1.7978, 124.5028)	2.50	0.0241

Rate ratios (RR) compared with NW/No EWG infant group from exponentiated beta coefficients. Wald confidence intervals for rate ratios. Z-score and FDR *p* value for original (non-exponentiated) beta coefficients.

Supplementary Table S21 Regression models for a maternal GDM and OW/OB interaction on each measured SCFA

SCFA	Group	Estimate	95% CI	*p* value
Butyrate	GDM	-0.148	(-0.41,0.11)	0.241
Butyrate	OW/OB	-0.050	(-0.31,0.21)	0.689
Butyrate	GDM:OW/OB	0.146	(-0.22,0.52)	0.419
Propionate	GDM	-0.139	(-0.54,0.26)	0.473
Propionate	OW/OB	-0.162	(-0.56,0.24)	0.405
Propionate	GDM:OW/OB	0.119	(-0.45,0.69)	0.668
Acetate	GDM	-2.101	(-9.76,5.56)	0.573
Acetate	OW/OB	-1.829	(-9.49,5.83)	0.623
Acetate	GDM:OW/OB	5.558	(-5.46,16.58)	0.304
Total SCFA	GDM	-2.389	(-10.38,5.6)	0.539
Total SCFA	OW/OB	-2.041	(-10.03,5.95)	0.599
Total SCFA	GDM:OW/OB	5.822	(-5.68,17.32)	0.302

p values and confidence intervals from a t-distribution with 19 degrees of freedom.

Supplementary Table S22 Regression models for a maternal OW/OB and EWG interaction on each measured SCFA

SCFA	Group	Estimate	95% CI	*p* value
Butyrate	EWG	0.116	(-0.16,0.39)	0.381
Butyrate	OW/OB	0.021	(-0.22,0.26)	0.854
Butyrate	EWG:OW/OB	-0.055	(-0.44,0.33)	0.768
Propionate	EWG	-0.069	(-0.48,0.35)	0.731
Propionate	OW/OB	-0.202	(-0.57,0.16)	0.263
Propionate	EWG:OW/OB	0.209	(-0.37,0.79)	0.462
Acetate	EWG	2.362	(-5.77,10.5)	0.550
Acetate	OW/OB	2.482	(-4.69,9.66)	0.478
Acetate	EWG:OW/OB	-4.042	(-15.48,7.4)	0.469
Total SCFA	EWG	2.410	(-6.09,10.91)	0.560
Total SCFA	OW/OB	2.302	(-5.19,9.8)	0.528
Total SCFA	EWG:OW/OB	-3.887	(-15.84,8.06)	0.504

p values and confidence intervals from a t-distribution with 19 degrees of freedom.

Supplementary Table S23 Spearman correlations between centered log ratio transformation of genus-level taxa counts and short-chain fatty acid measurements

Phylum	Genus	Acetate correlation	Propionate correlation	Butyrate correlation	Total SCFA correlation
Actinobacteria	*Actinomyces*	-0.004	-0.109	-0.105	-0.008
Actinobacteria	*Bifidobacterium*	-0.230	-0.510	-0.157	-0.255
Actinobacteria	*Corynebacterium*	-0.261	-0.439	-0.304	-0.289
Actinobacteria	*Rothia*	-0.225	-0.241	-0.149	-0.227
Actinobacteria	*Propionibacterium*	-0.032	-0.243	-0.137	-0.059
Actinobacteria	Unclassified *Coriobacteriaceae*	-0.223	-0.258	-0.088	-0.229

Actinobacteria	*Atopobium*	0.255	0.014	-0.183	0.240
Actinobacteria	*Collinsella*	-0.058	0.171	0.005	-0.067
Actinobacteria	*Eggerthella*	-0.081	-0.047	0.104	-0.057
Bacteroidetes	Unclassified *Bacteroidetes*	0.021	0.267	0.384	0.087
Bacteroidetes	Unclassified *Bacteroidales*	0.056	0.008	-0.077	0.025
Bacteroidetes	*Bacteroides*	0.082	0.122	0.024	0.115
Bacteroidetes	*Butyricimonas*	0.113	0.094	-0.038	0.137
Bacteroidetes	*Parabacteroides*	-0.072	-0.316	-0.149	-0.104
Bacteroidetes	*Porphyromonas*	0.113	0.094	-0.038	0.137
Bacteroidetes	*Prevotella*	-0.608	-0.273	-0.317	-0.552
Bacteroidetes	*Alistipes*	-0.114	-0.035	-0.009	-0.090
Firmicutes	Unclassified *Firmicutes*	0.187	0.415	0.394	0.221
Firmicutes	Unclassified *Bacilli*	0.129	0.200	0.076	0.095
Firmicutes	Unclassified *Bacillales*	-0.054	0.016	-0.025	-0.031
Firmicutes	*Gemella*	0.056	0.014	-0.323	0.053
Firmicutes	*Staphylococcus*	-0.133	0.049	-0.014	-0.114
Firmicutes	Unclassified *Lactobacillales*	-0.239	-0.123	0.234	-0.218
Firmicutes	*Enterococcus*	0.327	0.021	-0.055	0.259
Firmicutes	*Lactobacillus*	-0.080	-0.227	-0.213	-0.098
Firmicutes	Unclassified *Streptococcaceae*	0.267	0.366	0.110	0.270
Firmicutes	*Streptococcus*	-0.150	-0.019	-0.073	-0.135
Firmicutes	Unclassified *Clostridiales*	0.290	0.217	0.170	0.282
Firmicutes	Unclassified *Christensenellaceae*	-0.434	-0.410	-0.457	-0.454
Firmicutes	Unclassified *Clostridiaceae*	0.165	0.177	0.077	0.193
Firmicutes	*Clostridium*	0.168	0.257	0.397	0.184
Firmicutes	*Finegoldia*	0.225	0.199	0.062	0.222
Firmicutes	*Peptoniphilus*	0.155	0.124	-0.016	0.165
Firmicutes	Unclassified *Family-XIII-Incertae-Sedis*	0.086	-0.031	-0.371	0.061
Firmicutes	Unclassified *Lachnospiraceae*	-0.016	0.226	0.152	-0.001
Firmicutes	*Anaerostipes*	-0.002	0.060	-0.078	0.026
Firmicutes	*Blautia*	0.191	0.386	0.216	0.224
Firmicutes	*Dorea*	-0.117	0.000	-0.151	-0.094
Firmicutes	Unclassified *Peptostreptococcaceae*	0.011	0.142	0.101	-0.012
Firmicutes	Unclassified *Ruminococcaceae*	-0.078	0.059	0.271	-0.033
Firmicutes	*Anaerotruncus*	-0.236	-0.315	-0.338	-0.265
Firmicutes	*Faecalibacterium*	-0.008	0.172	0.067	0.050
Firmicutes	*Flavonifractor*	-0.075	-0.188	0.015	-0.078
Firmicutes	Unclassified *Erysipelotrichaceae*	0.305	0.250	0.260	0.319
Firmicutes	Unclassified *Selenomonadales*	0.178	0.240	0.032	0.201
Firmicutes	*Phascolarctobacterium*	0.067	0.233	0.032	0.098
Firmicutes	Unclassified *Veillonellaceae*	-0.357	-0.274	-0.107	-0.304
Firmicutes	*Megasphaera*	0.073	0.245	0.080	0.123
Firmicutes	*Negativicoccus*	-0.241	0.001	-0.268	-0.203

Firmicutes	*Veillonella*	-0.080	-0.089	-0.211	-0.106
Other	Unclassified Other	-0.139	-0.118	-0.023	-0.096
Proteobacteria	Unclassified *Proteobacteria*	-0.097	-0.133	-0.079	-0.099
Proteobacteria	*Thalassospira*	0.341	0.214	0.172	0.366
Proteobacteria	*Sutterella*	-0.137	-0.025	0.054	-0.087
Proteobacteria	*Neisseria*	0.330	0.313	0.022	0.283
Proteobacteria	*Bilophila*	0.169	0.134	0.121	0.126
Proteobacteria	*Desulfovibrio*	0.097	0.204	0.077	0.133
Proteobacteria	Unclassified *Gammaproteobacteria*	0.139	0.241	0.264	0.164
Proteobacteria	Unclassified *Enterobacteriaceae*	0.159	0.035	0.072	0.180
Proteobacteria	*Citrobacter*	0.230	0.105	-0.059	0.192
Proteobacteria	*Cronobacter*	0.155	0.079	0.262	0.175
Proteobacteria	*Enterobacter*	0.595	0.238	0.380	0.605
Proteobacteria	*Escherichia-Shigella*	0.061	0.347	0.347	0.123
Proteobacteria	*Klebsiella*	0.162	-0.158	-0.014	0.157
Proteobacteria	*Pantoea*	-0.020	-0.127	-0.281	-0.016
Proteobacteria	*Raoultella*	0.225	0.239	0.035	0.270
Proteobacteria	*Serratia*	0.147	0.100	0.154	0.172
Proteobacteria	*Haemophilus*	0.223	-0.032	-0.106	0.169
Proteobacteria	*Acinetobacter*	0.081	0.156	0.230	0.108
Proteobacteria	*Stenotrophomonas*	0.113	0.094	-0.038	0.137

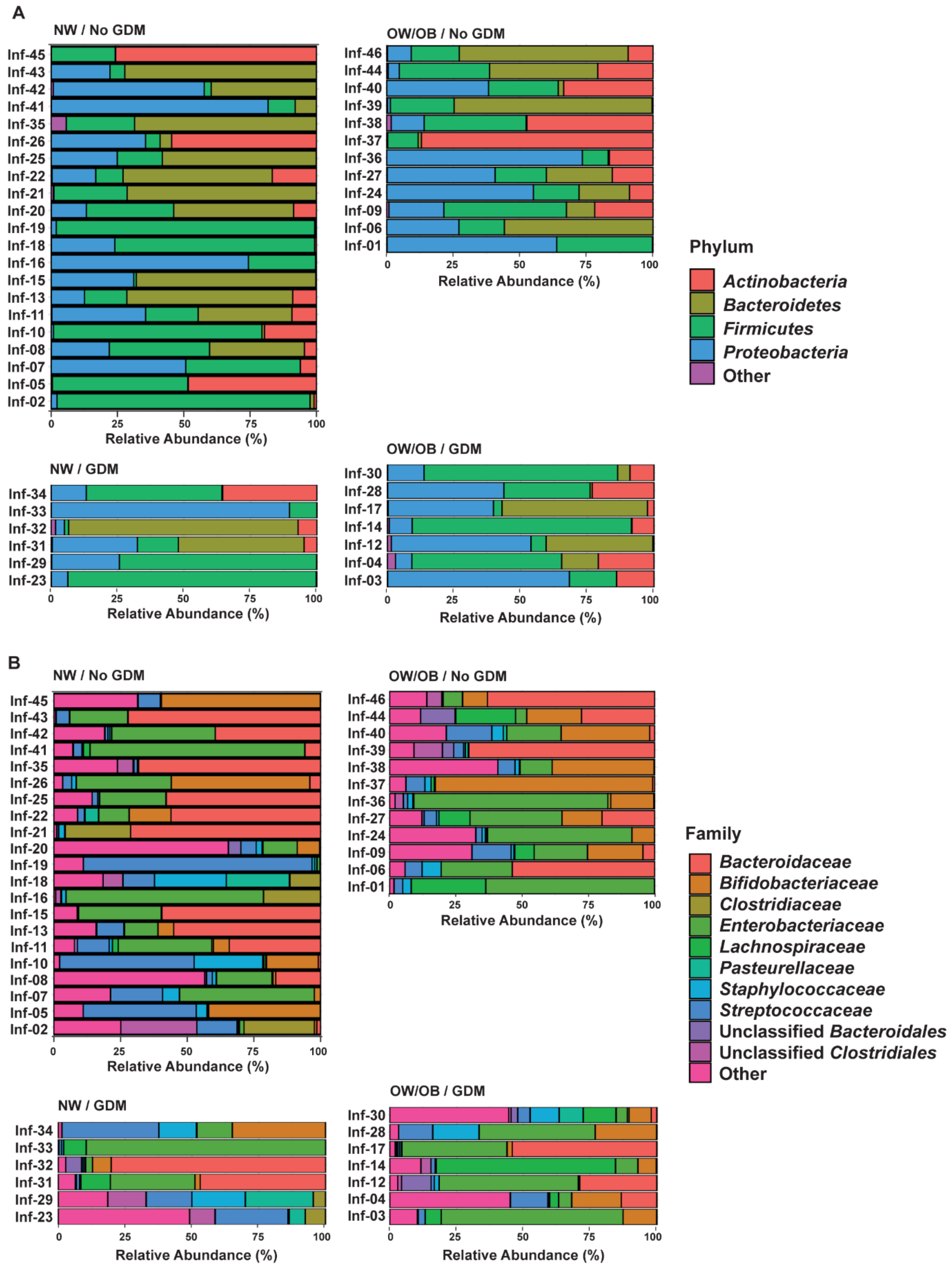

Supplementary Figure S1. The 10 most abundant taxa in the infant microbiota by GDM status. Relative abundance of microbiota taxa for each infant at the (**A**) phylum and (**B**) family levels. Phylum- and family-level taxa shown are the top 10 most abundant taxa with the remaining classified as Other. Relative abundance estimated using negative binomial regression on 16S rRNA gene amplicon sequences from stool samples, shown as percent of total. GDM, gestational diabetes; NW, normal-weight; OW/OB, overweight/obese.

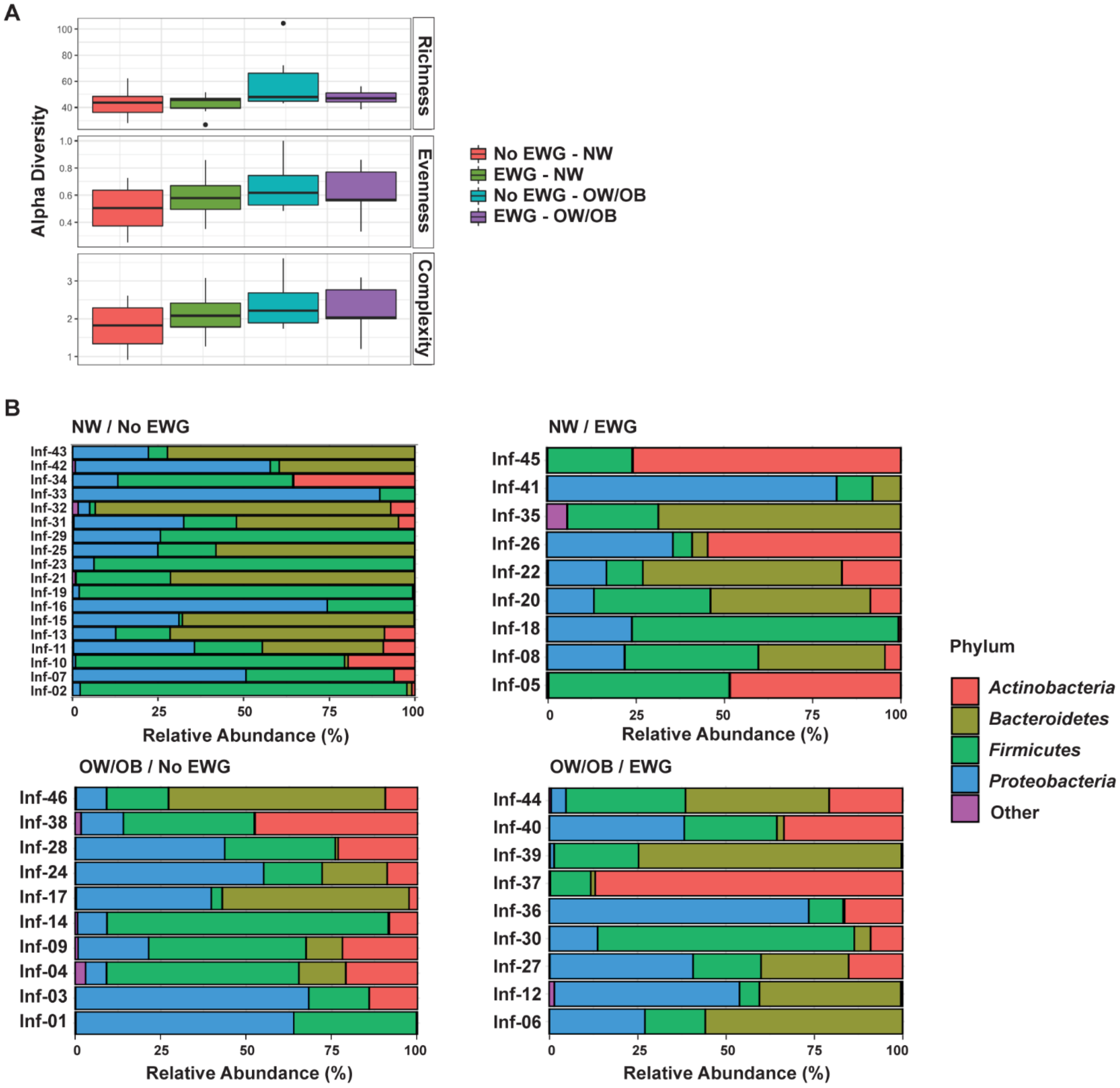

Supplementary Figure S2. EWG and maternal overweight/obesity alter infant gut microbiota. (**A**) Box plots of genera level alpha diversities richness (Chao1), evenness (Shannon $\frac{H}{H_{max}}$), and complexity (Shannon H) by weight gain and weight group. Black dots represent outliers within strata. **B**) Relative abundance of microbiome taxa for each infant at the phylum levels. Phylum-level taxa shown are the four most abundant with the remaining classified as Other. Relative abundance estimated using negative binomial regression on 16S rRNA gene amplicon sequences from stool samples, shown as percent of total. EWG, excessive gestational weight gain; NW, normal-weight; OW/OB, overweight/obese.

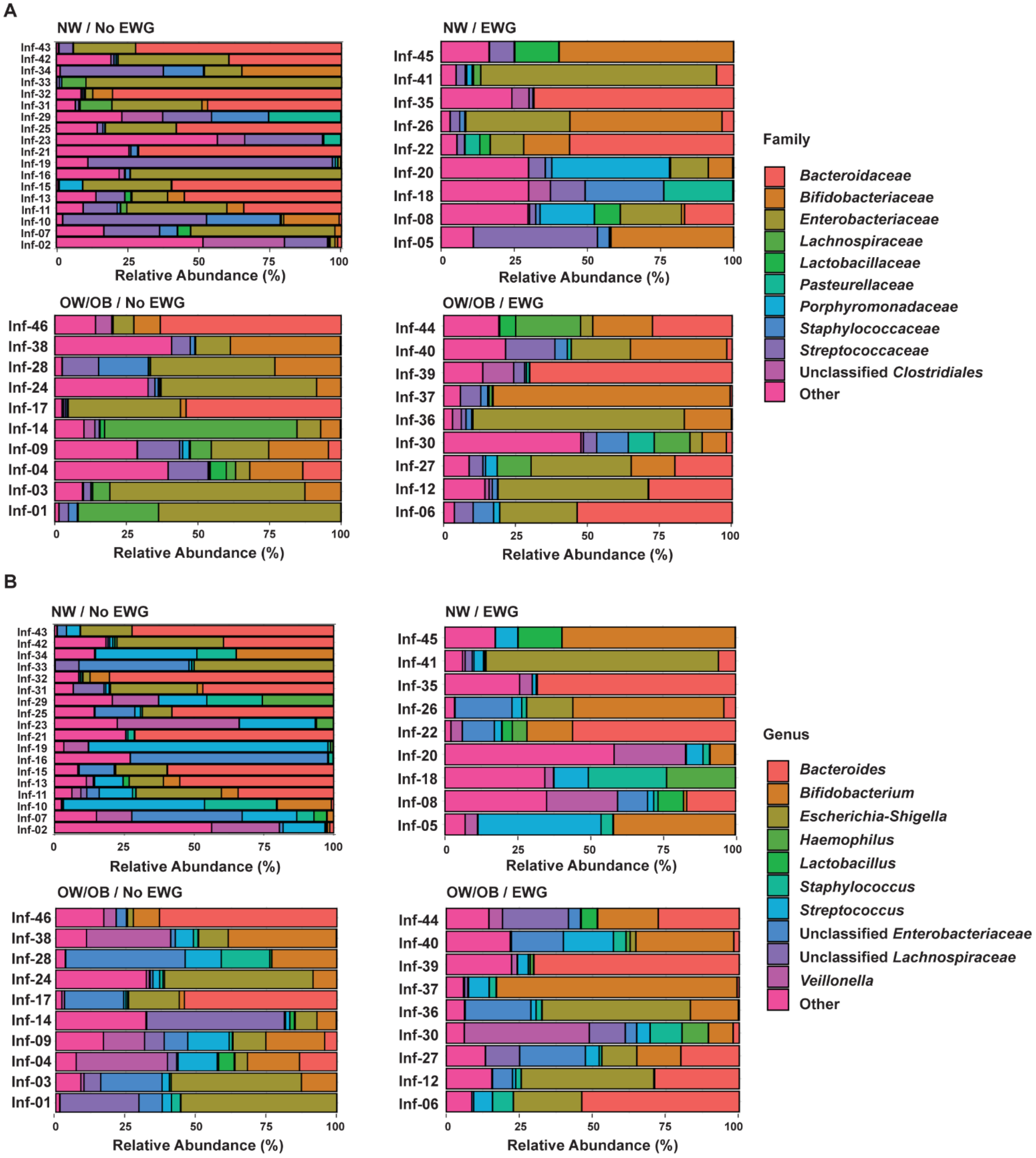

Supplementary Figure S3. The top 10 most abundant taxa in the infant microbiota by EWG status. Relative abundance of microbiota taxa for each infant at the family (**A**) and genus (**B**) levels. Family- and genus-level taxa shown are the 10 most abundant genera with the remaining classified as Other. Relative abundance estimated using negative binomial regression on 16S rRNA gene amplicon sequences from stool samples, shown as percent of total. NW, normal-weight; OW/OB, overweight/obese; EWG, excessive gestational weight gain.

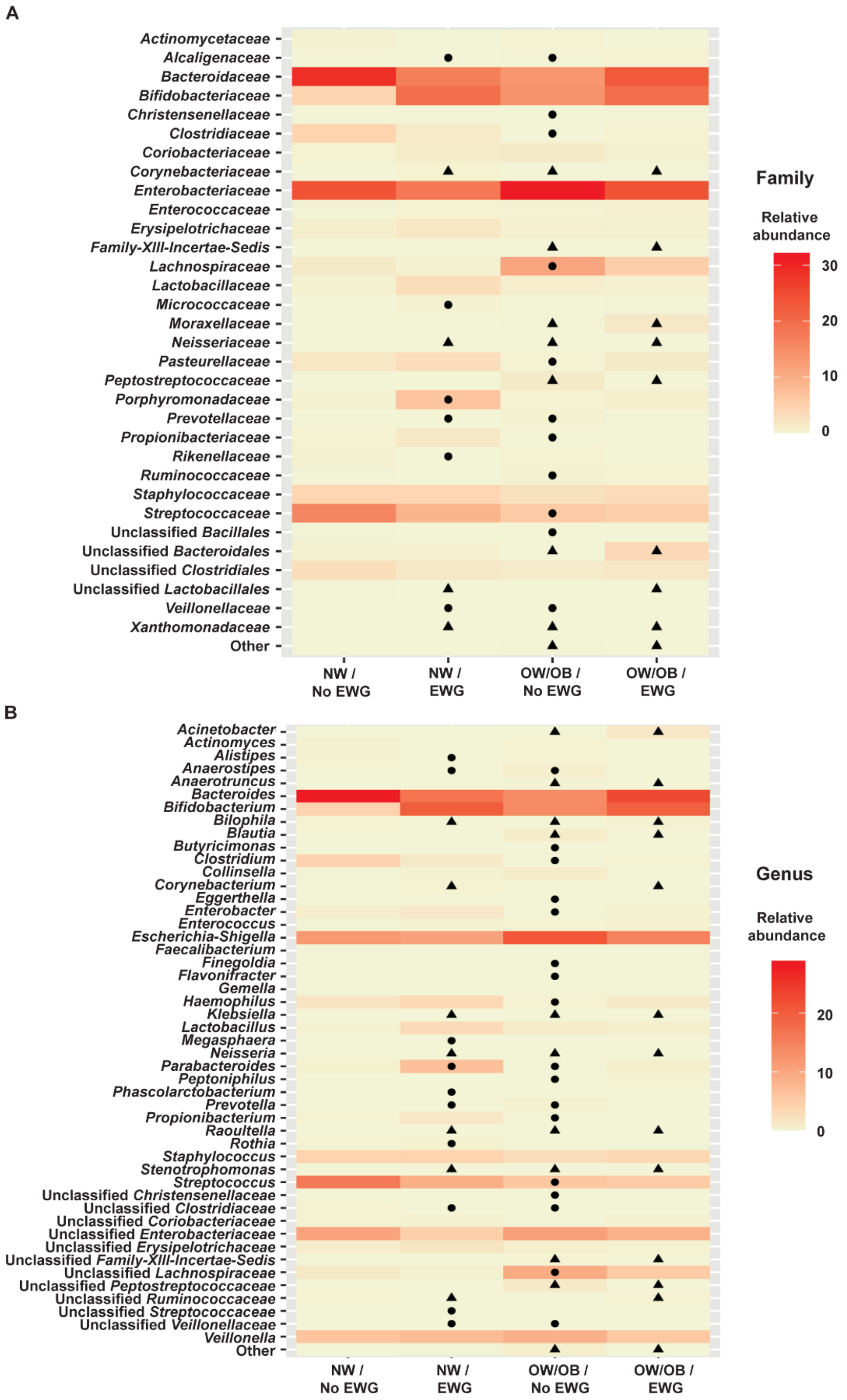

Supplementary Figure S4 Heat maps of estimated relationships between maternal phenotype and infant gut microbiota. Average taxa relative abundances (RA) are displayed by maternal phenotype with higher averages colored a deeper red. Cells with a shape inside indicate a significantly different (FDR-adjusted p value < 0.05) average RA from the NW/No EWG group. Triangles indicate significant differences from the interaction models and circles indicate differences from the additive models for both (**a**) family-level and (**b**) genus-level taxa.
